# Bayesian History Matching of Complex Infectious Disease Models Using Emulation: A Tutorial and a Case Study on HIV in Uganda

**DOI:** 10.1371/journal.pcbi.1003968

**Published:** 2015-01-08

**Authors:** Ioannis Andrianakis, Ian R. Vernon, Nicky McCreesh, Trevelyan J. McKinley, Jeremy E. Oakley, Rebecca N. Nsubuga, Michael Goldstein, Richard G. White

**Affiliations:** 1 Dept. of Infectious Disease Epidemiology, London School of Hygiene & Tropical Medicine, London, United Kingdom; 2 Dept. of Mathematical Sciences, Durham University, Durham, United Kingdom; 3 School of Medicine, Pharmacy and Health, Durham University, Durham, United Kingdom; 4 Dept. of Veterinary Medicine, University of Cambridge, Cambridge, United Kingdom; 5 School of Mathematics and Statistics, University of Sheffield, Sheffield, United Kingdom; 6 Medical Research Council/Uganda Virus Research Institute, Uganda Research Unit on AIDS, Entebbe, Uganda; University of Rochester, United States of America

## Abstract

Advances in scientific computing have allowed the development of complex models that are being routinely applied to problems in disease epidemiology, public health and decision making. The utility of these models depends in part on how well they can reproduce empirical data. However, fitting such models to real world data is greatly hindered both by large numbers of input and output parameters, and by long run times, such that many modelling studies lack a formal calibration methodology. We present a novel method that has the potential to improve the calibration of complex infectious disease models (hereafter called *simulators*). We present this in the form of a tutorial and a case study where we history match a dynamic, event-driven, individual-based stochastic HIV simulator, using extensive demographic, behavioural and epidemiological data available from Uganda. The tutorial describes history matching and emulation. History matching is an iterative procedure that reduces the simulator's input space by identifying and discarding areas that are unlikely to provide a good match to the empirical data. History matching relies on the computational efficiency of a Bayesian representation of the simulator, known as an *emulator*. Emulators mimic the simulator's behaviour, but are often several orders of magnitude faster to evaluate. In the case study, we use a 22 input simulator, fitting its 18 outputs simultaneously. After 9 iterations of history matching, a non-implausible region of the simulator input space was identified that was 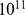 times smaller than the original input space. Simulator evaluations made within this region were found to have a 65% probability of fitting all 18 outputs. History matching and emulation are useful additions to the toolbox of infectious disease modellers. Further research is required to explicitly address the stochastic nature of the simulator as well as to account for correlations between outputs.

## Author Summary

An increasing number of scientific disciplines, and biology in particular, rely on complex computational models. The utility of these models depends on how well they are fitted to empirical data. Fitting is achieved by searching for suitable values for the models' input parameters, in a process known as calibration. Modern computer models typically have a large number of input and output parameters, and long running times, a consequence of their increasing computational complexity. The above two things hinder the calibration process. In this work, we propose a method that can help the calibration of models with long running times and several inputs and outputs. We apply this method on an individual based, dynamic and stochastic HIV model, using HIV data from Uganda. The final system has a 65% probability of selecting an input parameter set that fits all 18 model outputs.

## Introduction

Complex computer models (hereafter called simulators) are now being used in many scientific disciplines and are becoming increasingly common in basic science, climate modelling, communicable and non-communicable disease epidemiology and public health [1]–[6]. The simulators' utility for prediction and planning relies on how well they are calibrated to empirical data and how well they can be analysed to assess the validity of their predictions [7], [8].

Simulators can be calibrated using a multitude of approaches. Simple ‘goodness of fit’ methodologies, such as least squares, are often used, however these approaches are difficult to apply to high-dimensional and computationally expensive individual-level simulators. More rigorous statistical techniques have been developed, usually based around the concept of a likelihood function. These techniques are very flexible, and can be used to fit a wide variety of simulators, ranging in complexity. Nonetheless, implementing likelihood-based inference techniques for complex simulators is challenging, particularly when considering large-scale, missing or partially observed data. Recent advances that have been usefully applied in the field of dynamic epidemic modelling include maximum likelihood via iterated filtering [9]; data augmented and/or reversible-jump Markov chain Monte Carlo (MCMC; [10]–[13]) and stochastic differential equations [14]. However, these systems can sometimes become mathematically or computationally intractable, leading to the development of various approximation techniques [15], [16].

A common theme in approximation methods for dynamic simulators is to replace dependence on the likelihood with outputs from simulator runs, since although a model's likelihood may be intractable, running the simulator is straightforward. These approaches can be implemented in various ways, for example: embedded in a particle filter [17]; using Approximate Bayesian Computation [18]–[20]; or using pseudo-marginal methods [21]. Another technique that could be applied in this field is particle MCMC [22].

Despite the variety of calibration methods, their application to the analysis of complex simulators is lacking. A systematic review of cancer simulators found that of 131 studies only two thirds (87) provided any information on what methods were employed. Of these only about a third (27) used a formal goodness of fit measure (two used likelihood-based methods and 25 distance-based metrics, such as least squares or chi-square) [23]. The remainder did not state how the simulators were calibrated or used visual inspection to assess how well the simulator described the data. Similarly, a systematic review of simulators of HIV transmission in men who have sex with men found only 18% of the 115 simulators had been formally calibrated to data and remarkably that calibration had become less common over time [24].

One of the key reasons that complex simulator calibration is uncommon is that most formal methods (including distance-based and likelihood-based measures) require that simulators are run many times [25]. This poses a considerable problem for complex simulators that require several minutes or even hours for the evaluation of a single scenario, making most of the above calibration methods utterly impractical. The problem is compounded for stochastic simulators because hundreds or thousands of realisations are required for each scenario. Current standard methods for formal sensitivity and uncertainty analysis [26] are also impractical for complex simulators because of the heavy computational burden [27]. Simulator simplification, although desirable, is not appropriate if a complex simulator is required to satisfactorily address the research question and it increases the probability of simulator inadequacy [25]. As the number of simulator parameters increases, the number of runs required for an adequate exploration of the parameter space increases rapidly. Robust fitting and uncertainty analysis of complex simulators with dozens of parameters is often impossible, even with increasing computer power and advances in parallelisation. Another important aspect of the calibration of complex models that remains unaddressed in the epidemiology literature is that of model discrepancy [25], [28]–[30]. This represents an upfront acknowledgement of the limitations of the complex model and helps tailor the search for acceptable input parameters by providing a more rigorous and realistic definition of match quality between the model outputs and observed data (see section ‘History matching’).

In this work we present a novel method based on Bayesian history matching, emulation and model discrepancy, that is designed to address all of the above issues while simultaneously avoiding unnecessary complexity. This method has the potential to greatly improve the calibration of complex infectious disease simulators. We present this in the form of a tutorial (section ‘Methods’) and a case study where we history match a dynamic, event-driven, individual-based stochastic HIV simulator, using extensive demographic, behavioural and epidemiological data from Uganda (section ‘Results’). The online supplementary material includes the details required for building an emulator and a simulation study that demonstrates the performance of history matching on synthetic data.

## Methods

### Motivation

A major issue that affects the calibration algorithms discussed so far arises from simulators that are slow to evaluate. Although computers are becoming increasingly powerful, running times of hours or days are not uncommon (as modellers tend to develop more complex simulators to exploit increased computing power). This can render any calibration algorithm that relies on a large number of simulator evaluations utterly impractical. Another issue is that modern simulators tend to have a large number of inputs and outputs and the task of matching several outputs while varying a large number of inputs simultaneously can be very intensive computationally. Both of these conditions can be addressed with history matching and emulation.

History matching [28] is designed to identify the set of inputs that would give rise to acceptable matches between the model outputs and the observed data. It has three characteristics that distinguish it from most calibration methods. Firstly, many calibration algorithms (for example Bayesian MCMC) attempt to make full probabilistic statements about the input values that are most likely to match the simulator's output to the empirical data. This represents a challenging and computationally intensive task, involving complex and frequently intractable calculations. Critically, often such detailed calculations are unwarranted as the complex model is not thought to be an accurate enough representation of reality to justify them. History matching instead provides a more tractable calculation involving expectations and variances, that is often of primary interest to modellers. Secondly, history matching works by excluding parts of the input space that are unlikely to provide a good match. These parts of the space are known as *implausible*. The third characteristic is that the implausible space is not excluded all at once, but in iterations of the process, known as *waves*. As a result, the *non-implausible* space (i.e. the complement of the implausible space), shrinks at each iteration of the process.

The above characteristics give some desirable properties to history matching. First, the calculations involved are far more efficient and straightforward to implement. Second, the exclusion of implausible space is possible without considering the full set of inputs and outputs simultaneously, thus reducing the burden of high dimensionality. For example, if the simulator fails to match one output for a particular input value, then this value is implausible regardless of the other outputs' behaviour. This should be compared to fully probabilistic approaches (for example full Bayesian MCMC or maximum likelihood methods) which attempt to model how likely an input is, usually using a likelihood function, thus representing a far more complex calculation that must use all outputs and all observed data and information simultaneously. Third, as the volume of non-implausible space shrinks with consecutive waves, often to a tiny fraction of the original, the simulator's behaviour typically becomes more predictable and smooth, as the range of the inputs is significantly smaller. Once this point is reached, handling the full set of inputs and outputs is normally more manageable: at this point more detailed probabilistic calibration methods can be employed if necessary (see section ‘Posterior sampling’). Finally, it is possible that a simulator is incapable of matching the observation data, due to either incorrect modelling assumptions, poor error specification, or coding errors. History matching can identify this condition by characterising all the input parameter space as implausible, whereas alternative methods will always attempt to return a posterior distribution, regardless of how well, if at all, the simulator fits the data.

A long established method for handling computationally expensive simulators is to first construct an *emulator*: a statistical model of the simulator that can be used as a surrogate [31]. The simulator is first run at a manageable number of input values, to provide training data to build the emulator. The emulator will give a joint probability distribution of the simulator outputs for any set of input values, and the distribution can be used both to provide estimates of the outputs, and quantify uncertainty in the estimates. Building an emulator will involve some computational effort in obtaining the training data (the simulator runs) and fitting the emulator to the data. However, once built, the emulator can provide estimates (with a quantification of uncertainty) of the simulator output near instantaneously, even for very large numbers of inputs. Emulators enable rapid exploration of high dimensional input spaces, and have been used within fully probabilistic calibration [25], [32], [33], including simulators with high dimensional output [34].

Emulators can be used within history matching if the simulator is computationally expensive, as is the case in this work. History matching together with emulation has been successfully applied across a range of scientific disciplines including galaxy formation simulations ([35], [36] or for an overview see [37]), oil reservoir models [28], [38], systems biology models [39], [40], climate models [41] and rainfall runoff models [30].

History matching is a method designed for reducing the simulator's input space but is not designed to make probabilistic statements about the inputs, such as producing posterior distributions. Thus, it can be seen as a pre-calibration method or as a calibration method but in the broader sense. We would assert that for many situations involving model development and assessment, the results of a history match are all that are required by the modeller. When specifying the initial input ranges, we may have substantial uncertainty about what the acceptable input values are, so that the acceptable region of the input space (that would contain say the posterior distribution) is a tiny proportion of the initially specified input space, and thus hard to discover. The iterative nature of history matching and the fact that it discards the implausible space instead of looking for input values that are close to the empirical data simplify significantly this task. It is also important to bear in mind that alternative ‘probabilistic’ calibration methods would most likely struggle with a model of the complexity and input-output dimensionality such as the one studied here. Therefore, should one wish to probabilistically calibrate a well tested and accurate simulator, it is still advantageous to greatly reduce the input space under consideration first, using history matching as a precursor.

We continue this tutorial by describing how history matching is set up (section ‘History matching’), and we then present the procedure of history matching (section ‘Procedure’) along with a toy example that illustrates the fundamental concepts. The tutorial then proceeds with two more technical sections, one containing details on how an emulator is built (section ‘Emulation’), and another describing an essential component of history matching, the implausibility measure (section ‘Implausibility measure’). Finally, we present an approximate method for drawing samples from the simulator's posterior distribution (section ‘Posterior sampling’).

### History matching

**Figure 1:**
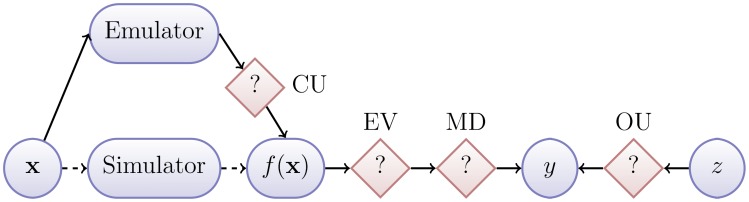
History matching. The physical process 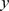 is observed via 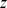 and described by the simulator output 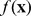. The simulator is substituted by the emulator for computational efficiency. The question marks indicate the various sources of uncertainty present in the system.

History matching assumes the existence of a physical process 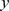 that is measured through observations 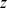 (Fig. 1). The acquisition of observations 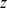 takes place with finite accuracy and introduces some uncertainty, which we term observation uncertainty (OU). History matching also assumes the existence of a simulator (computer model) that attempts to describe the process 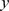. The simulator has 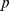 inputs (parameters) 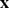, assumed to be continuous 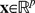. We consider a stochastic simulator: a simulator which when run twice at the same value of 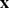 can produce different outputs.

We suppose that the simulator output consists of a vector of 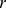 quantities, which we denote with the vector 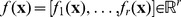. To represent the stochastic nature of the simulator, if we keep the input vector 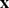 fixed and run the simulator 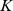 times, we would observe, for the 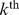 run, with 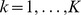: 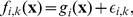
(1)where 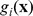 is the mean value of the 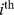 output (if the simulator were to be run repeatedly at the same input value 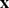), and 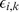 is a random variable with expectation 0.

We suppose that the physical process 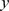 corresponds, to some level of accuracy or tolerance, to a realisation of the simulator output 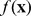, at some particular input, rather than the mean output 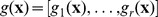. In our search for non-implausible inputs, we need to take into account the variability of 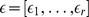 around 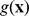. We refer to this term as Ensemble Variability (EV).

As mentioned earlier, the calibration of complex simulators can be infeasible if the calibration method depends on a large number of simulator evaluations that take considerable time to complete. For this reason, we rely on a statistical model of the simulator, known as an emulator, which is trained using a relatively small number of simulator runs and which we use to provide an estimate of 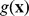 in a fraction of the time required for a simulator run. The emulator represents our beliefs about the 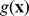 at all, yet to be evaluated inputs 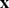, and our uncertainty about such values. The fact that the simulator (code) is not evaluated for every possible value of 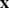, creates another source of uncertainty, which we term Code Uncertainty (CU) and is quantified via the emulator.

There is one final source of uncertainty, which is important though perhaps the most difficult to consider. Due to our incomplete understanding of the process 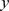 and our inability to model all of its aspects, we do not believe the simulator to be a perfect representation of reality [28]. This has three implications for calibration. Firstly, an input that gives a good match to historical data will not necessarily give a good prediction of future data; the simulator may be overfitted. Secondly, an input that does *not* give a good match to one physical output quantity may *still* give a good prediction of another physical output quantity, if the simulator models some quantities more accurately than others. Thirdly, if the inputs are physically observable quantities (that could, in principle, be learnt independently of the simulator), failing to account for an imperfect simulator can lead to overconfident posterior distributions that are centred on the wrong values [42].

We refer to this final source of uncertainty as Model Discrepancy (MD) [25]. Incorporating model discrepancy protects against overfitting, ensures that we do not exclude possible values of future observations, when the exclusion would be unwarranted, is necessary for inferring the true values of simulator inputs, when the notion of a true input value is clearly understood, and is required for making realistic forecasts ([35], [43]).

In summary, we link the observation of the physical process to the best simulator input, which we denote by 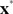 via 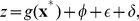
(2)where 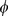 is a vector of errors representing observation uncertainty, 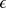 is a vector of errors representing ensemble variability, 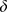 is a vector of errors representing model discrepancy, and 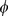, 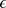, 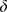 and 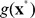 are judged to be independent [25].

### Procedure

**Figure 2:**
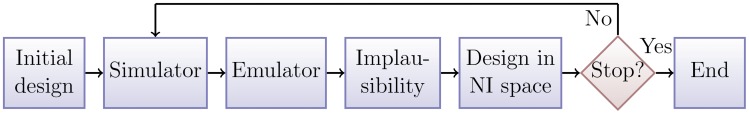
History matching workflow. The simulator is evaluated at carefully selected design points. Its output is used to train the emulator, which, with the help of the implausibility measure, determines the parts of the input space which are non-implausible (NI). The simulator is then evaluated at set of design points from the non-implausible space and the procedure is repeated until one or more stopping criteria are met.

Fig. 2 shows a typical history matching workflow. The first step is the selection of a number of input values (design points) at which the simulator is run. The initial inputs are chosen using a maximin Latin hypercube design [44], which generates uniformly distributed points, but also aims to fill the entire input space, by maximising the minimum distance between the points generated. The number of points 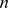 in this initial design depends on the available computational resources. A very approximate rule of thumb is to use at least 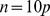 for training the emulator and 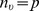 points for validation [45].

Once the initial design space, 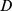, is specified, the simulator is run at the selected points 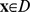. Following the notation set out in equation 1, we construct 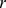 separate emulators: one for the mean of each output 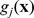, with 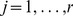. For the *j*-th output, the training data takes the following form. We choose the training inputs 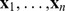 and for each input value 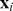, we run the simulator 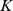 times, to generate observations 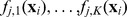. We then calculate the sample mean and variance of the simulator runs at input 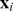:
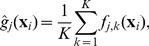
(3)




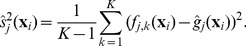
(4)


The training data point for input 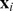 is then 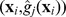, where 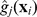 is an estimate of 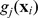. The number of runs (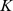) per input point are determined by the simulator's complexity and the available computational power. A relatively large number of repetitions (e.g. 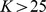) will ensure that the error in the estimate is approximately normally distributed with expectation 0 and variance 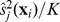 even if the individual 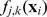 terms are not normally distributed. Once we have built the 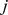
th emulator, we can efficiently obtain an expected value of 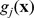 and variance for any 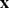, in particular for input values where we have *not* run the simulator. We denote this expectation and variance by 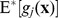 and 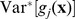, where the superscript 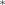 indicates that the expectation and variance refer to code uncertainty: the fact that 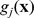 is an uncertain quantity. Other approaches of emulating separately the simulator's mean and variance are also possible [33], [39], [40]. It should be noted that often some of the outputs are difficult to emulate in the first few waves, in which case we would emulate a subset of the 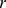 outputs initially, emulating the remaining outputs in later waves, when the process becomes easier due to the reduced size of the input space. More detail on emulation is given in section ‘Emulation’.

**Figure 3:**
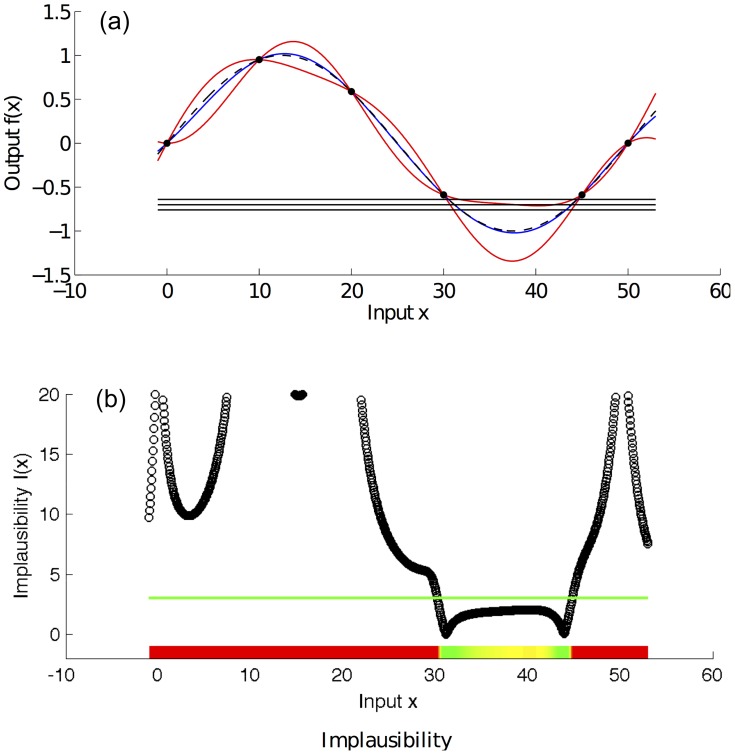
Example emulator and implausibility for toy simulator [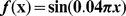]. Panel (a) shows an emulator of the toy simulator 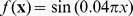 (black dashed line). The value of 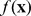 is considered unknown apart from six points where the simulator is run and are represented by the black dots in the figure. The blue line is the emulator's posterior mean, and the red lines represent its posterior uncertainty (95% CI). The 3 horizontal lines represent the empirical data (mean value and 95% CI) that we use to history match the simulator. Panel (b) shows the implausibility for the emulator and the empirical data shown in panel (a). The implausibility is large when the emulator's posterior mean is far from the empirical data, relatively to the uncertainties present in the system (observation and code uncertainty in this case). The horizontal green line is an implausibility cut-off, which determines whether an input 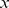 is implausible or not.

Fig. 3(a) shows a simple example of a one dimensional emulator. The (toy) simulator used is the deterministic function 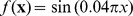. Because the simulator is deterministic, it holds that 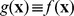. The value of 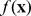 is considered unknown apart from the six points 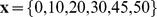 where the simulator is run and are represented by the black dots in the figure. The blue line is the emulator's posterior mean, and the red lines represent its posterior uncertainty (95% CI). The 3 horizontal lines represent the empirical data (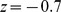) and the 95% CI (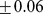) that we use to history match the simulator.

The next step involves choosing an implausibility measure and defining its various components. The implausibility is an essential element of history matching and is a measure that estimates whether the input 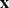 is likely to result in an output that will match the observations. It essentially weighs the difference between 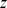 and 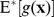 with all the uncertainties that are present in the system. The implausibility is large when the emulator's posterior mean is far from the empirical data, relative to the uncertainties present in the system (observation and code uncertainty in this case). An analytical description of how the implausibility can be formulated is provided in section ‘Implausibility measure’.

Fig. 3(b) shows the implausibility for the emulator and empirical data from Fig. 3(a). The horizontal green line is an implausibility cut-off, which determines whether an input 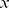 is implausible or not. The implausibility plot shows that a match between the simulator's output and the empirical data is unlikely to be found for values of 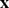 smaller than 30 and larger than 45.

With the emulators and the implausibility measure at our disposal we can then carry out two key functions of history matching: the first is to sample the non-implausible space and study its distribution. This can reveal input combinations that can lead to acceptable matches, correlations between inputs and detailed insight into the model's structure. The second function is the creation of a design that is space filling over the current non-implausible space, which will be used to run the simulator in the next wave (iteration) of history matching.

The simplest method for sampling the non-implausible space, is to draw samples uniformly from the entire input space and reject those that fail the implausibility criteria. This method is computationally straightforward, but it can become inefficient when the non-implausible space is a tiny fraction of the original space, which is often true, especially in later waves. A method for solving this problem using an evolutionary Monte Carlo algorithm was proposed in [46]. In this paper, we propose a simpler but also effective method. Suppose that in wave 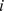 we have a number of non-implausible points 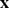. For each of these, we draw 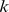 samples from a 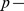
variate normal distribution that is centered on the value of the generating point. The 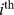 wave implausibility is then evaluated on the new samples and the variance of the normal distribution is selected so that a small percentage of them (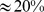) are non-implausible. The low acceptance rates should ensure that the new samples are sufficiently different from the old ones. This method can efficiently generate an adequate number of data points that can be used in subsequent waves.

A subset of the non-implausible samples drawn are then used to run the simulator and repeat another wave of history matching. The code or emulator uncertainty decreases with each iteration for the following reasons. At each wave, the emulators are only constructed over a smaller region of input space compared to the previous wave, and therefore the mean of the simulator outputs are usually smoother functions of the input parameters and hence easier to emulate accurately. Also there is a higher density of simulator runs in the new reduced input space, which again leads to improvements due to the Gaussian process part of the emulator as described in section ‘What is an emulator?’. There may be additional benefits due to active variable selection as discussed in [35], [37], and new outputs that were previously difficult to emulate may now become available. A major reason for the power of the history matching approach described here, is due to the above improvements to the emulation process at each wave, allowing the iterative exploration of complex input spaces.

**Figure 4:**
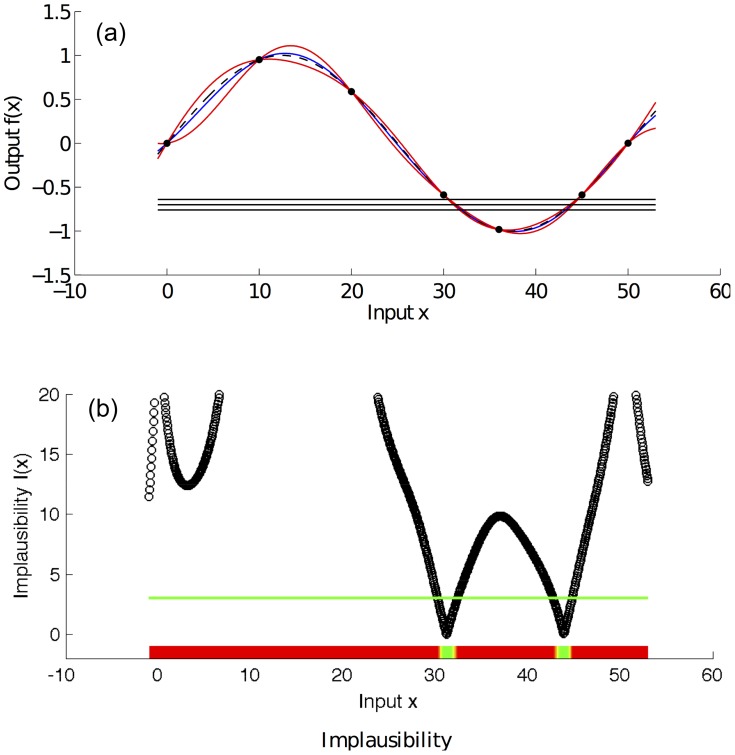
Second history matching wave for the toy simulator 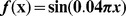. Panel (a) shows an emulator of the toy simulator 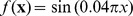 (black dashed line). The value of 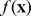 is considered unknown apart from seven points where the simulator is run and are represented by the black dots in the figure. The blue line is the emulator's posterior mean, and the red lines represent its posterior uncertainty (95% CI). The 3 horizontal lines represent the empirical data (mean value and 95% CI) that we use to history match the simulator. Panel (b) shows the implausibility for the emulator and the empirical data shown in panel (a). The implausibility is large when the emulator's posterior mean is far from the empirical data, relatively to the uncertainties present in the system (observation and code uncertainty in this case). The horizontal green line is an implausibility cut-off, which determines whether an input 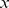 is implausible or not.

Fig. 4 shows the second wave of history matching for the running example of this section. The simulator was run for the non-implausible value of 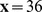 and this point was included in the training data. Note how the emulator's posterior variance has decreased in the region of interest. Consequently, the non-implausible region has shrunk dramatically, indicating that a match can only be found for 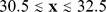 and 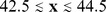, where indeed the function 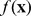 takes values between −0.8 and −0.63.

The procedure can continue with more waves until one or more stopping criteria are met. One such criterion is when all the input space is deemed non-implausible, meaning that the simulator cannot match the observations given the current error specifications. In this case one would then vary the size of the model discrepancy to determine how large it would have to be to obtain a match: a very large model discrepancy would suggest that the simulator is inadequate as a model for the physical process in question, and that further model development is required.

Another stopping criterion occurs when the emulators have a posterior variance smaller than the remaining uncertainties in the system (the observation uncertainty, model discrepancy and the ensemble variability), as this condition implies that the non-implausible space contains acceptable matches and is unlikely to decrease in size in the next iteration, unless the remaining uncertainties in the system can be revised and decreased as well. Here we would check the acceptable matches against any other outputs that were not used in the emulation process. A final condition for stopping could be the fact that the simulator runs obtained in the current wave are close enough to the empirical data and we do not wish to continue any further. In these two cases, we would investigate the sensitivity and robustness of the non-implausible region obtained from the history match, to alterations in the observation uncertainties and model discrepancy [47].

### Emulation

#### What is an emulator?

An emulator represents our beliefs about the behaviour of an unknown function. In this application, where the simulator is stochastic, the unknown function is taken to be the mean of the 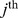 output of the simulator denoted as 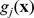. This function is observed (with error in the stochastic case) only at a limited number of points, 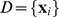 for 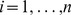, known as design points. An emulator also has a number of parameters 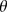 that determine its characteristics, (e.g. smoothness), the estimation of which is referred to as *training*. The emulator provides a probability distribution for the mean of the simulator's output at an untested input point 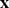, conditional on the simulator runs 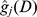 and an estimate of the parameters 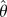, i.e. 
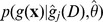
.

The emulators we consider in this paper have the form: 
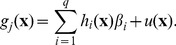
(5)


The first part is a regression term, where 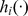 are known deterministic functions of 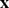 and the 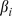 are the regression coefficients. The second part of the emulator is a stochastic process, known as a Gaussian process, which is stationary, with zero mean and constant variance [25], [48].

For a number of discrete points 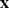, the model of equation 5 implies that 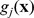 will follow the joint normal distribution 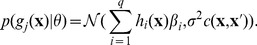
(6)


The summation term represents the emulator's mean, 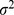 is the variance parameter and 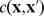 is the correlation function of the Gaussian process. The emulator's parameters are the triplet 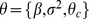, with 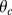 being some parameters specific to the correlation function 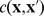 [49]. Details about the correlation function and the specifics of building an emulator including the choices of 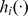 are provided in section ‘Extensions’ and in S1 Text.

*Training.* One way of training the emulator is by maximising the likelihood 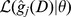 to obtain point estimates 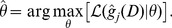
(7)


An alternative method is to define a prior distribution for the parameters 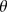 and marginalise them in the Bayesian sense, either analytically or numerically. In this work, we marginalise analytically the parameters 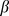 and 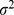. Since the marginalisation of the parameters 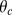 is not analytically tractable, we use point estimates, as the computational simplicity of this approach was found to outweigh the benefits of numerical marginalisation. Such use of point estimates has been successfully applied in [25], [35] and for a discussion see [32]. Another successful approach is not to view the correlation lengths 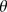 as parameters to be estimated or marginalised over, but instead to view them as direct prior quantities, the values of which can be asserted a priori using a variety of heuristic arguments, as described in [35].

*Validation*. After training the emulator it is necessary to perform some diagnostics that ensure that the emulator is sufficiently accurate. [50] provide a number of such diagnostics, such as the Mahalanobis distance, the analysis of prediction errors, the pivoted Cholesky decomposition etc., that can diagnose most failings of an emulator and suggest possible remedies. In the present case study we reserved approximately 20 model runs at each wave which were used to check the emulators' predictive performance.

#### Extensions

What we described above is a basic procedure for building an emulator. Some useful extensions to the basic methodology are given below.

*Mean and correlation function.* The regression functions 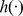 can have a very simple form, such as a constant 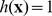 or a simple polynomial 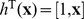, with 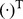 denoting vector transpose. However, they can be arbitrarily complicated or have a form that explains the data best. Examples of more complex polynomials and relevant selection procedures can be found in [35]. Similarly, there is a wide variety of correlation functions that can be used, depending on beliefs about the simulator's smoothness and differentiability [48].

*Transformations.* In many cases, the Gaussian process model of equation 5 might be better suited to a transformed version of the simulator's output. Applying a transformation that makes the output more Gaussian can benefit the emulation process. Mapping all the input ranges to the [0,1] range is another common practice that helps fitting and interpreting the Gaussian process part of the emulator.

*Multiple outputs.* The simplest way of emulating a multi-output simulator is via an array of independent univariate emulators. This is the approach we take in this paper. However, if one is interested in correlations between outputs, multi-output emulators can take these into account [34], [51]–[53].

### Implausibility measure

#### One dimensional implausibility

The implausibility measure is a function of the input 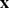, and returns a large value if it is unlikely that evaluation of the simulator at input 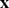 would result in an acceptable match between the simulator's output and the empirical data, given all current uncertainties. For one output of the simulator it can be written as
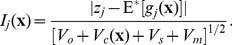
(8)


The term 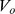 represents the variance associated with the observation uncertainty. The 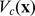 term represents the code uncertainty as given by the emulator and is hence set to 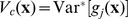. 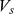 is the ensemble variability representing the stochastic nature of the simulator. For simplicity, it can be assumed to be constant over the input space and set equal to the mean of the sample variances obtained in the current wave: 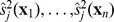. In the case study described in section ‘Results’ we make the more conservative choice of setting 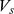 equal to the 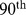 percentile of the sample variances. A more advanced but also more complex method is described in [39], [40], whereby the variance of the simulator is also emulated allowing 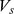 to be made an explicit function of 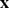. Finally, the model discrepancy term is represented by 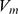. Ideally, its exact form is elicited from model experts, who are asked to quantitatively assess the simulator inaccuracy for different outputs, due for example to known defects, missing components or insufficient modelling detail [35]. An alternative approach is to incorporate discrepancy parameters within the simulator, with the effect that the elicitation task is broken down into considering individual sources of discrepancy within the model ([30], [54]).

In this work, we take the much simpler approach of considering it equal to 10% of the variance of the simulator output training data 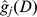 obtained at each wave. This conservative choice is made for illustrative purposes, so that all of the different sources of uncertainty will contribute to the final answer. A full analysis would involve a combination of elicitation and sensitivity analysis regarding the specific judgements made [47]. It should be noted that the effect of incorporating any amount of discrepancy is simply to expand the set of inputs that are classified as non-implausible. Typically, at the final wave, we will still have a reasonable proportion of inputs classified in both the implausible and non-implausible sets, and the simulator user can investigate the effects of changing the simulator discrepancy variance (or even setting it to 0) without difficulty [47]. (If, however, all inputs are classified as non-implausible at the final wave, inflating the discrepancy variance at this stage would not change the classification.)

A large value of 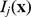 would indicate that despite the uncertainties present in the system, our prediction about the simulator's output for 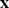 is so far from the observed value 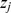, that the simulator is very unlikely to match the data at that particular point. However, we still need to find a value 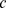 for 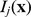 that will act as a cut off, such that all 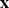 for which 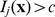 will be deemed implausible. Such a cut off can be provided by Pukelsheim's 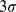 rule [55]. This rule states that *any* continuous unimodal distribution contains at least 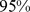 of its probability mass within a distance of 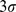 from its mean, where 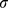 is its standard deviation: an extremely powerful and general result. If we consider that for a fixed 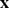 the random quantity 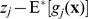 has a unimodal distribution, and suppose that 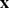 is an input that matches the simulator's output to the empirical data, then according to Pukelsheim's rule we would have 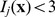 for at least 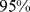 of the time. The above argument provides a way of assessing the magnitude of the one dimensional implausibility, without being forced into making full distributional assumptions for all quantities involved in equation 8.

#### Multi-dimensional implausibility

In the case of a multi-output simulator, the simplest approach is to construct one implausibility per output, i.e. 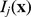, for 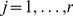 and consider the maximum implausibility at 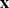


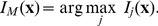
(9)


Considering the second (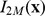) or third (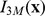) highest implausibility for each 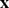 and applying appropriate cut-off values to each measure is another option, as is implemented in [35].

The implausibility also comes in a multivariate form which is given by 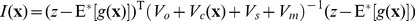
(10)where 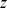 and 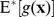 are vectors of length 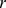, and the quantities 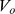, 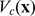, 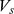 and 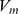 are all now covariance matrices of dimension 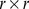. Often it can be difficult both to specify the full covariance structure for 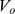 and 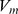, and to calculate it for 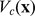 and 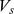 (which would require more advanced multivariate emulators). A common approach is to assume the outputs are uncorrelated and hence that 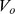, 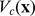, 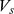 and 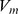 are all diagonal matrices, with the univariate variance that corresponds to the 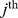 simulator output (given in the denominator of equation 8) being placed in the 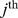 position in the diagonal. Cut-off values for 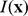 can be found for example from a suitably high percentile (e.g. 95%, 99%) of the chi-squared distribution with 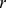 degrees of freedom, as 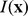 can be seen as a squared sum of variance normalised random variables (see [35]).

Let us finally note that the above forms of implausibility (e.g. 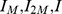) along with their appropriately chosen cutoffs, can be used simultaneously, and require a point 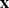 to have a low score in all the above measures so as to be considered non-implausible.

### Posterior sampling

By design, history matching excludes parts of the input space that produce poor fits to the empirical data and leads to, where possible, the generation of a large number of runs that give acceptable fits to the observed data. Often this is sufficient for model analysis, e.g. to help understand the biological or epidemiological mechanisms underlying the physical system, and model development to determine the next improvement to the mathematical model and hence the next extension to the computer code. However, history matching does not provide full Bayesian posterior distributions for the uncertain quantities of interest (for example, the input parameters). Were it the case that full posterior distributions are required, for say a highly accurate, well tested and well understood epidemiology model, we now show how the results of history matching, specifically the identification of the final non-implausible region of input space (which should enclose the posterior distribution), can be used to obtain samples from the posterior. Such information from the posterior can be used for many tasks such as comparing competing models, and also when making forecasts into the future (a typical use of epidemic models).

Let 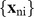 be the non-implausible samples from the last wave of history matching. We formulate the following proposal distribution 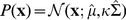
(11)which is a multivariate normal distribution, with mean chosen to be the sample mean 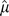 of the non-implausible samples 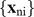 and with variance chosen to be 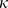 multiplied by the sample variance-covariance matrix 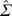 of the non-implausible samples. The constant 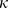 is used to inflate the variance of the non-implausible samples, so that the method explores a larger part of the input space, as this was found to improve the sampling process. We define the approximate likelihood of the input parameters 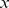 with observed data 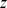 as
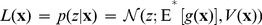
(12)with 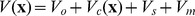. This follows from equation 2 combined with the additional assumptions that the observation error 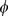, model discrepancy 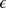, ensemble variability 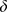 and the emulator representation of 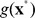 are all normally distributed. Such normality assumptions for 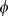 and 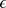 are very common (see for example [25]), and for the emulator this is simply equivalent to having sufficient runs to ensure the emulator's t-distribution can be viewed as approximately normal, as is described in S1 Text. The assumption of normality of the ensemble variability (which is directly analogous to the assumptions made in standard regression) may not be justified in some cases, however the above approximations can still be accurate when 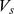 is smaller that the other variance components so that their total 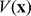 can still be considered approximately normal. More detailed modelling of 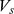 is of course possible [39], [40], and more complex distributions can be specified, however this would only be warranted if the epidemiology model was judged to be a sufficiently accurate mimic of reality that the details of the distribution of its ensemble variability were physically meaningful. In the case study described in section ‘Results’, this was definitely not thought to be the case.

We assume that the prior 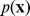 is constant over the final non-implausible region found from the history match. This is in many cases reasonable as the volume of this region is often many orders of magnitude smaller that the original input space, and hence any prior that was not strongly informative would likely be approximately constant over such a small space. Note however, that the algorithm below can be easily adapted for any prior, by a suitable scaling of the weights.

The algorithm proceeds as follows: we first use the proposal distribution 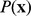 to generate a number of samples 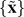. We then calculate a weight for each sample as 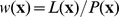. Finally, we draw the desired number of posterior samples 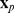 from the set of 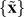, with a probability defined by the weights 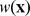. Provided the weights are reasonably well behaved, this will generate direct draws from the approximate posterior 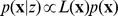.

## Results

### Simulator and empirical data

This case study was based on a research project that explored the effects of partnership concurrency (overlapping sexual partnerships) on HIV transmission in Uganda [56]. The simulator used in the research study, named Mukwano, was a dynamic, stochastic, individual based computer model that simulates heterosexual sexual partnerships and HIV transmission. In an individual based micro simulation model, the life histories of hypothetical individuals are simulated over time in a computer program. Each individual is represented by a number of characteristics, of which some remain constant during simulated life (e.g. gender and date of birth), whereas others change (e.g. HIV status). Changes in personal characteristics result from events such as the start and the end of sexual relationships. These events are stochastic: if and when an event occurs is determined by Monte-Carlo sampling from probability distributions. To generate model outcomes for a simulated population, the characteristics of the simulated individuals are aggregated. The simulator had been fitted to empirical data in a number of scenarios by eye and by changing the values of inputs, which control various demographic, behavioural and epidemiologic characteristics of the simulated population [56].

Births, deaths, partnership formation and dissolution and HIV transmission were modelled using time-dependent rates. At birth, simulated individuals were assigned to one of two sexual activity groups (‘high activity’ and ‘low activity’) and to one of two concurrency groups (‘high concurrency’ and ‘low concurrency’). Each sexual activity group had associated male and female sexual contact rates, which determined the rate at which individuals formed new partnerships, which were of two types (‘short duration’ and ‘long duration’).

For the present case study, we apply our history matching and emulation methodology to the primary baseline scenario from [56], rather than fitting ‘by-eye’. Twenty behavioural and two epidemiologic inputs were varied, including a mixing parameter, which determines the tendency for individuals to preferentially form partnerships with people in their own activity group, and an input which determines the duration of the long and short duration partnerships. The behavioural inputs are permitted to take different values in each of three calendar time periods. This enables sexual behaviour to vary over time. The full list of the 22 simulator inputs and their original plausible ranges is shown in Table 1.

**Table 1:** Simulator input parameter description and ranges.

Number	Input description	Abbr.	Min.	Max.
1	Proportion of men in the high sexual activity group	*mhag*	0.01	0.5
2	Proportion of women in the high sexual activity group	*whag*	0.01	0.5
3	Mixing by activity group [ 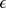 ]	*mag*	0	1
4	High activity contact rate (risk behaviour 1) [partners/yr]*	*hacr1*	0	10
5	Low activity contact rate (risk behaviour 1) [partners/yr]*	*lacr1*	0	2
6	Start year for risk behaviour 2	*sy2*	1986	1992
7	High activity contact rate (risk behaviour 2) [partners/yr]*	*hacr2*	0	10
8	Low activity contact rate (risk behaviour 2) [partners/yr]*	*lacr2*	0	2
9	Start year for risk behaviour 3	*sy3*	1998	2002
10	High activity contact rate (risk behaviour 3) [partners/yr]*	*hacr3*	0	10
11	Low activity contact rate (risk behaviour 3) [partners/yr]*	*lacr3*	0	2
12	Mean HIV transmission probability per sex act during primary stage of infection (mean of male to female and female to male transmission probabilities)	*atp*	0	1
13	Ratio of male to female/female to male transmission probabilities	*rtp*	1	3
14	Proportion of low activity men in high concurrency group	*lmhc*	0	1
15	Proportion of low activity women in high concurrency group	*lwhc*	0	1
16	Male concurrency parameter in high concurrency group (risk behaviour 1)	*mchc1*	0	1
17	Female concurrency parameter in high concurrency group (risk behaviour 1)	*fchc1*	0	1
18	Male concurrency parameter in high concurrency group (risk behaviour 2)	*mchc2*	0	1
19	Female concurrency parameter in high concurrency group (risk behaviour 2)	*fchc2*	0	1
20	Male concurrency parameter in high concurrency group (risk behaviour 3)	*mchc3*	0	1
21	Female concurrency parameter in high concurrency group (risk behaviour 3)	*fchc3*	0	1
22	Duration of long-duration partnerships [years]	*dlp*	5	20

These define the input parameter space over which the history match search is performed.

*The simulator input parameters that codetermine partnership formation. The actual rate of partnership formation in the simulator will vary from this due to adjustment for concurrency and partnership balancing.

The simulator was history matched using 18 demographic, behavioural and epidemiologic outputs that include male and female population sizes, and male and female HIV prevalences at three time points. They also include a number of outputs that ensure that the prevalence and incidence of monogamous and concurrent sexual partnerships in the simulator closely matched the data from the empirical population. The empirical data were collected from a rural general population cohort in South-West Uganda. The cohort was established in 1989 and currently consists of the residents of 25 villages [57]–[59]. Every year, demographic information on the cohort is updated, the population was tested for HIV, and a behavioural questionnaire was conducted. In 2008, this included questions that allowed the prevalence of monogamous and concurrent short duration and long duration partnerships to be calculated. All 18 simulator outputs and their calibration targets are shown in table 2. The intervals given for each of the outputs represent the limits for an acceptable match, and we consider them as 95% confidence intervals. Therefore, their mean value is used to define the value of the observed data 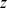, and their difference is chosen to represent 4 times the square root of the observation error 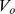.

**Table 2:** Description of simulator outputs and limits defined as an acceptable match.

Number	Output description	Abbr.	Min.	Max.
1	Population size in 2008 (male)	*psm*	2986	3650
2	Population size in 2008 (female)	*psf*	3374	4124
3	Average male partnership incidence in 2008 (partners/year)	*ampi*	0.4	0.489
4	HIV prevalence in 1992 (male)	*p92m*	0.084	0.112
5	HIV prevalence in 1992 (female)	*p92f*	0.096	0.124
6	HIV prevalence in 2001 (male)	*p01m*	0.07	0.09
7	HIV prevalence in 2001 (female)	*p01f*	0.083	0.107
8	HIV prevalence in 2007 (male)	*p07m*	0.06	0.084
9	HIV prevalence in 2007 (female)	*p07f*	0.093	0.119
10	Point prevalence of men with 1 long duration partnership in 2008 (%)	*m1l*	34.62	42.31
11	Point prevalence of men with 1 short duration partnership in 2008 (%)	*m1s*	10.86	13.27
12	Point prevalence of men with 1 partnership (either type) in 2008 (%)	*m1*	37.83	46.24
13	Point prevalence of men with 2+ long duration partnerships in 2008 (%)	*m2l*	3.38	4.13
14	Point prevalence of men with 2+ short duration partnerships in 2008 (%)	*m2s*	1.69	2.07
15	Point prevalence of men with 2+ partnerships (any combination) in 2008 (%)	*m2*	8.66	10.59
16	Point prevalence of women with 2+ long duration partnerships in 2008 (%)	*w2l*	0.85	1.03
17	Point prevalence of women with 2+ short duration partnerships in 2008 (%)	*w2s*	0.42	0.52
18	Point prevalence of women with 2+ partnerships (any combination) in 2008 (%)	*w2*	2.17	2.65

The simulator was run on a high performance cluster with 240 nodes. The run time for a single simulation varied between 10 minutes and 3 hours. One emulator evaluation on a standard laptop took approximately 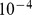 seconds, a speed ratio in the order of 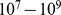. A simulation study with synthetic data and a smaller version of Mukwano, which demonstrates the validity of history matching is included in the online supplementary material.

### Wave 1

The first wave of emulators was trained using a 220 point maximin Latin hypercube design [60]. A separate 20 point Latin hypercube was used for generating the validation data. The simulator was run 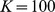 times at each design point, to allow the estimation of 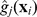 and 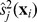 with sufficient accuracy, for subsequent use as plug-in estimates.

Univariate emulators were built and successfully passed the validation tests mentioned in section ‘What is an emulator?’ for 16 out of the 18 outputs. The emulators used a third order polynomial mean function (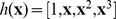) and the Matérn correlation function. If a more complex set of polynomials 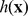 was used, various linear model selection methods can be employed as in [35], [38]. The logit transformation was used for the outputs that by definition lay in the [0,1] interval. Similarly, all inputs were mapped to the [0,1] (unit interval) to facilitate the interpretation of the 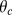 parameters. Emulators for outputs 8, 9 did not validate in the first attempt and rectifying this would have involved further efforts such as generating more design points, or using more detailed mean functions. These two outputs were left out of the first wave analysis, but this poses no problem to history matching, because the exclusion of non-implausible space does not require considering all outputs at once (unlike a likelihood based approach). All we require is a subset of outputs that will sufficiently reduce the non-implausible space at the current wave. In subsequent waves, the behaviour of these two outputs became more regular, and they were included in the analysis.

Drawing a large ensemble of non-implausible points for studying their distribution and for proposing the design for wave 2 was the next step in the analysis. 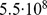 points were drawn from a 22 dimensional uniform distribution in [0,1] and the implausibility was evaluated for each one of them. The implausibility used in the first wave was the maximum implausibility (equation 9) with the uncertainties specified as described in section ‘One dimensional implausibility’. On a regular laptop, the evaluation would have taken approximately 5 hours. However, since the whole process was very easy to parallelise, the evaluation was done on a 240 node cluster, and was completed in less than 5 minutes. Without the use of emulators and considering that the simulator was around 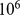 times slower, and it was evaluated 100 times for each scenario, this procedure would have taken around 1000 years! From the proposed samples only 21644 passed the implausibility test, implying that the volume of non-implausible space at this wave is 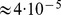 of the original input space.

**Figure 5:**
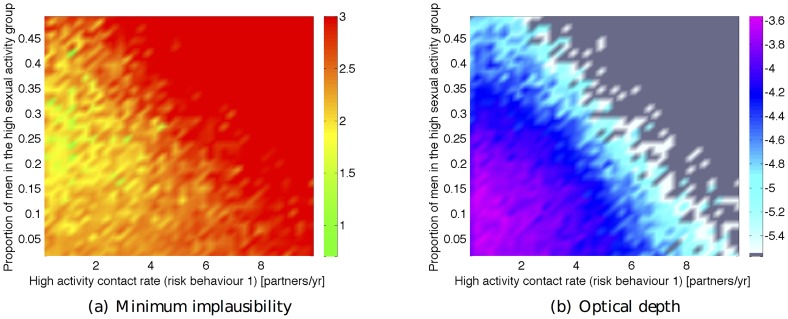
Minimum implausibility (a) and optical depth (b) plots for inputs 1 and 4 in wave 1. Minimum implausibility plots show an estimate of the minimum implausibility for different values of pairs of inputs. Optical depth plots show an estimate of the 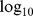 probability of encountering a non-implausible point for different values of pairs of inputs.

Visualising the distribution of the non-implausible points is conveniently done via minimum implausibility and optical depth plots [35], such as the ones shown in Fig. 5. To construct the minimum implausibility points, two inputs 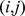 are first selected and a rectangular grid covering their range is formed. The non-implausible points are placed in the respective bin of this grid, according to the value of their 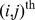 element. The plot shows the minimum implausibility value among all points in a given bin. Assuming a sufficiently large number of non-implausible samples, this kind of plot provides an empirical estimate of the minimum implausibility that can be expected if we were to fix inputs 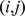 to a particular value, and hence shows locations in 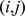 space that can be ruled out as implausible, irrespective of the choices of all the 20 other inputs. The optical depth plots are constructed in the same fashion, but instead of displaying the minimum implausibility per grid point, they display an empirical estimate of the probability of encountering a non-implausible point for a given set of values for inputs 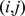. This estimate can be obtained from the ratio of non-implausible to total drawn points per bin. They therefore provide an estimate of the (higher-dimensional) depth of the non-implausible region, conditioned on the inputs 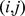.

**Figure 6:**
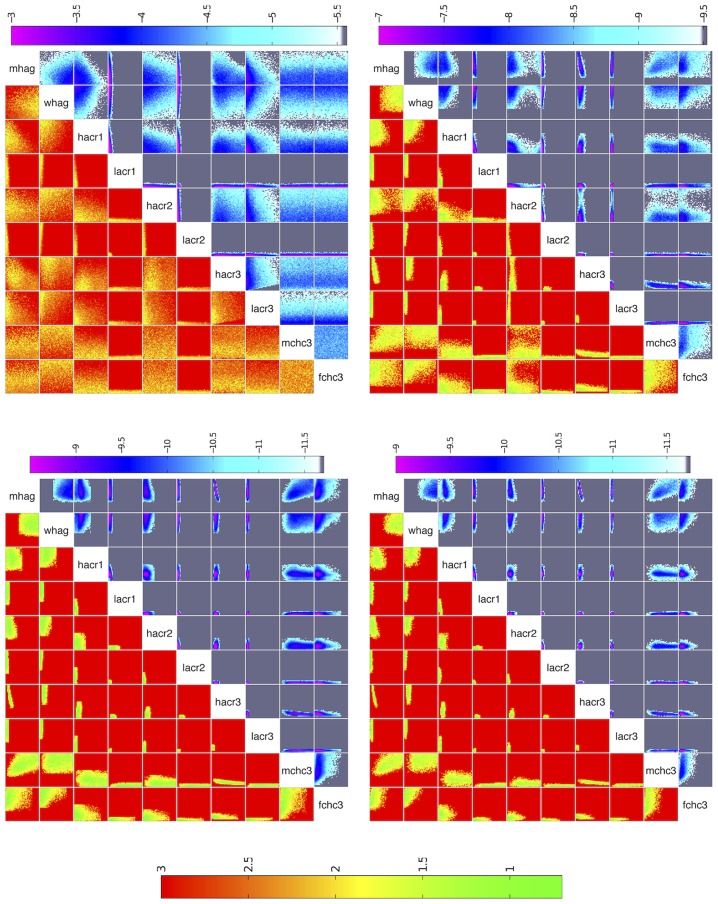
Minimum implausibility (below and left of diagonal) and optical depth plots (above and right of diagonal) for 10 key inputs for waves 1,4,7,9. Minimum implausibility plots show an estimate of the minimum implausibility for different values of pairs of inputs. Optical depth plots show an estimate of the 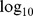 probability of encountering a non-implausible point for different values of pairs of inputs.

Fig. 5 shows the minimum implausibility and depth plots for the percentage of men in high sexual activity group (*mhag*) and the contact rate for high activity group in the first period (*hacr1*). This figure shows that a match was unlikely to be found if both inputs take a large value. Fig. 6(a) shows the implausibility and depth plots for 8 of the most active inputs in wave 1. It is noticeable that the contact rates for the low activity groups in the first two periods (*lacr1, lacr2*), only lead to matches when they take a relatively small value (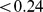). Finally, correlation patterns appear to emerge between a few input pairs, such as between inputs *mhag* and *hacr1*, and *mhag* and *hacr3*.

### Waves 2–10

The design for wave 2 can be obtained by uniformly drawing 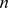 points from the non-implausible space of wave 1. To ensure that these points are sufficiently separated from each other, several *n*-point designs were drawn and we then chose the one with the maximum minimum (maximin) distance between its points. The simulator was run at the selected design points and the whole process was repeated up to the end of wave 9, where it was decided to stop as 50% of the wave 9 simulator runs were non-implausible (see section ‘Implausibility of the simulator runs’). A further 500 wave 10 runs were carried out to provide us with an estimate of the probability that a wave 9 non-implausible point would actually result in an non-implausible simulator run. This estimate was 65% (see section ‘Implausibility of the simulator runs’).

During the course of the 9 waves, some modifications were made to the history matching apparatus, which are described below: for the first three waves only the maximum implausibility was used, with a cut-off value of 3. Due to reduced rejection rates, from wave 4 onwards an input 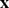 was deemed non-implausible if it passed all 3 tests: 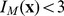, 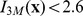 and 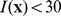. The latter cut off was chosen as it approximately represents the 95% critical value of a chi squared distribution with 18 degrees of freedom. For the first 2 waves, 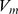 was set to a tenth of the simulator output variability as described in section ‘One dimensional implausibility’. The minimum between this value and 3 times the observation uncertainty was chosen for waves 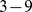, an illustrative choice, thought to be conservative with respect to the opinion of the expert, which resulted in further increases in the rejection rates at each wave. Finally, as the emulator uncertainty was fairly large in the first 3 waves, it was decided from wave 4 onwards, to have 500 simulator runs per wave and also include in the training of the emulators any simulator runs from previous waves that were deemed as non-implausible.

Table 3 shows the acceptance rates for all 9 waves. The acceptance rate for wave 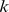 is defined as the proportion of non-implausible samples in wave 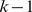 that remain non-implausible in wave 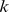, multiplied by the acceptance rate of the 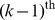 wave. Thus, the acceptance rates are a measure of the original input space shrinkage, since they represent the proportion of points drawn at random from the original input space that will be non-implausible after 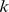 waves.

**Table 3:** Acceptance rates for the 9 waves, expressing the probability that an input 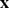 drawn at random from the original input space passes the 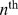 wave's implausibility test.

Wave 1	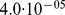
Wave 2	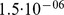
Wave 3	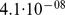
Wave 4	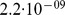
Wave 5	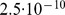
Wave 6	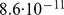
Wave 7	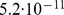
Wave 8	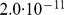
Wave 9	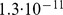

At wave 9, the non-implausible region is a tiny fraction (10^−11^) of the original input space, implying that we have learnt a large amount from the history matching process.

At wave 9, the non-implausible region is a tiny fraction (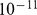) of the original input space, implying that we have learnt a large amount from the history matching process.

For the first 5 waves, direct sampling from the input space and evaluation of the various implausibilities could generate sufficient numbers of non-implausible samples at a reasonable computational cost. The exclusion of large portions of inputs *lacr1, lacr2, hacr3, lacr3* from as early as the first few waves (e.g. see Fig. 6(b)) helped speed up the sampling process. From wave 6 onwards however, this direct sampling method was proving inadequate. To overcome this problem and as discussed in section ‘Procedure’, 10000 non-implausible samples from wave 5 were perturbed to generate 20 samples each using a zero mean multivariate normal distribution. The distribution's variance was chosen such that the rejection rate of the new samples using the wave 5 implausibility was around 80%. These samples were then subjected to the wave 6 implausibility, which resulted in approximately 10000 wave 6 non-implausible samples. The same procedure was followed for the subsequent waves. The implausibility plots for waves 4, 7, 9 are shown in Figs. 6(b–d), which visualise the reduction of the non-implausible space in successive waves.

Apart from visualising the non-implausible space, implausibility plots can also reveal correlations that can exist between inputs: Fig. 6(d) shows that for the proportion of men in the high activity group (*mhag*) and for the high activity group contact rate in the third period (*hacr3*), non-implausible runs are unlikely to be found outside a narrow range of values. This is because if both are high (or low), the average male partnership incidence in 2008 will be unacceptably high (or low). It is only when one takes a high value and the other a low one, or both take intermediate values, that the partnership incidence output will fall within the acceptable limits.

A similar, although less correlated, relationship can be seen between the proportion of men in the high activity group (*mhag*) and the high activity group contact rates in the first and second periods (*hacr1*, *hacr2*). In these cases, when either both inputs are high or both are low, then the sexual behaviour outputs and the trend in HIV prevalence outputs cannot be fitted simultaneously. This is because if both are high then the very high levels of sexual activity during earlier years necessitate a very low HIV transmission probability in order to fit the earlier HIV prevalence output(s), which results in either the later HIV prevalence output(s) being too low, and/or the partnership incidence in 2008 being too high. A similar argument applies if both are inputs are low. It should be stressed that such insight into the model's structure and the required trade-offs between sets of inputs, can be readily obtained from a history match analysis, without the need for a more detailed study.

### Sensitivity of the implausibility measure

At the end of a history match, it is possible to experiment by reducing the uncertainty terms in the implausibility measure and re-evaluate the non-implausible space. This exercise can indicate which terms are most dominant. It is important to note however, that increasing the uncertainty terms at a latter wave is not possible, as the space that would have been retained at earlier waves had we used larger values for them cannot be recovered and the results will be inaccurate: one would have to start all over again. For this reason it is important to start history matching using our largest estimates for the uncertainties we believe are present in the system, as these can be reduced later but they cannot be increased.

Table 4 shows what percentage of the input space calculated as non-implausible would be found as implausible if the respective uncertainties were to be decreased by the percentages shown in the first column. Improving the EV estimates could increase the rejection rates, as it is shown in the 4th column of the table. We should note however, that, unlike the other terms, EV cannot become arbitrarily small, as it represents the stochastic variability in the simulator's output. Revising the observation errors could also help rejecting more space, followed by building more precise emulators (i.e. less CU) and revising the model discrepancy term.

**Table 4:** Percentage of the non-implausible space at wave 9 that would be calculated as implausible if the uncertainty shown in the first row was reduced by the amount shown in the first column.

	OE	CU	MD	EV
%	19.8	11.8	10.7	54.8
%	45.4	24.9	21.9	91.4

### Implausibility of the simulator runs

**Figure 7:**
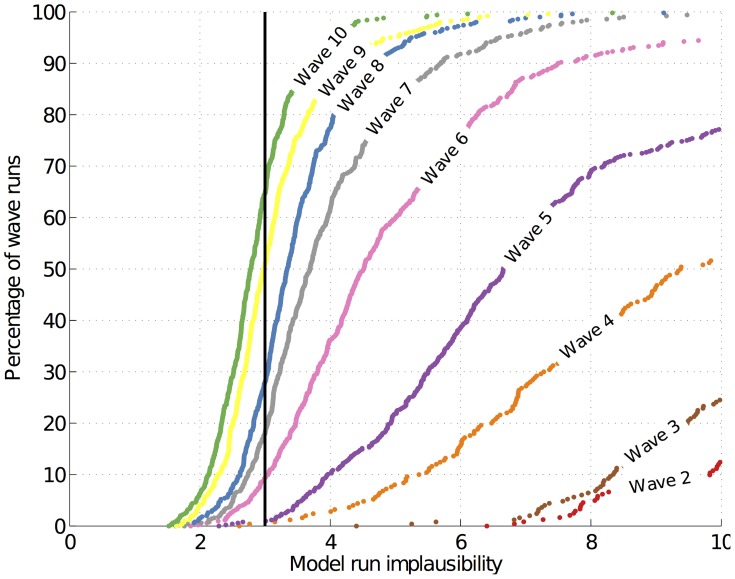
Cumulative distribution function of simulator run implausibility 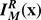 by waves. Each line represents the percentage of each wave's simulator runs with an 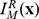 less than the value indicated by the x-axis.

In this section we examine the fit of the simulator output to the empirical data in successive waves. We first define the implausibility for one output of the actual simulator runs as 
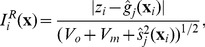
(13)with 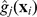 and 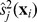 the run sample mean and variance as defined in equations 3 and 4. We also define the maximum implausibility of a run at input 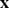 as 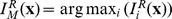. Note that this version of the implausibility does not include code uncertainty, as the simulator has been evaluated at 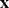 and that the ensemble variability is estimated directly from the simulator run (and so we may now describe runs as ‘acceptable’ if their implausibility is low). Fig. 7 shows the implausibility of the simulator runs in successive waves. In wave 9, 50% of the runs were non-implausible while in wave 10, the non-implausible (or acceptable) runs were 65% of the total number of runs, all coming from a region that is a tiny fraction (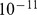) of the original input space. As we were then in a position to generate large numbers of acceptable runs from the non-implausible region with a 65% acceptance rate, the history match was concluded.

**Figure 8:**
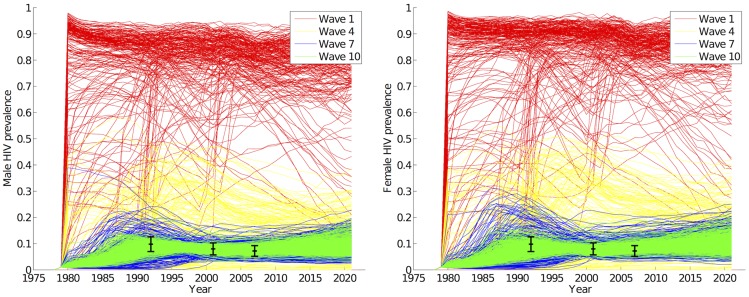
Simulator output (male and female HIV prevalence) in waves 1, 4, 7 and 10. The black lines show the average observed HIV prevalence with 95% credible ranges.

**Figure 9:**
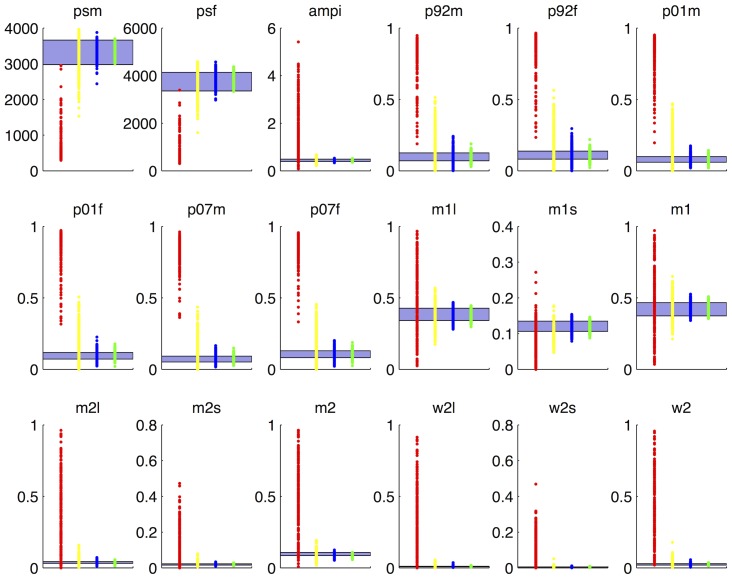
Convergence of the simulator's output to the empirical data with successive waves of history matching. Each of the 18 panels shows the range of the target data (horizontal region) and the simulator's output in waves 1 (red), 4 (yellow), 7 (blue) and 10 (green) (left to right along the x-axis).

Fig. 8 shows the time evolution of male and female HIV prevalence from simulator runs from four different waves. The empirical data are the male and female HIV prevalences in 1992, 2001 and 2007. The crosses represent the average observed values for each year and the credible ranges (error bars) represent 2 standard deviations calculated from the sum of the observation uncertainty and the model discrepancy for wave 9. Since we assume that the physical process 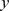 is one realisation of the simulator (barring the model discrepancy) and not its mean output, Fig. 8 shows the run of each scenario 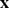 that best matched the empirical data. Note the wave 1 runs entirely miss the targets and that the majority of the wave 10 runs pass through them. Finally, Fig. 9 shows all 18 simulator outputs in waves 1, 4, 7 and 10 and their convergence to the empirical data.

### Posterior samples

**Figure 10:**
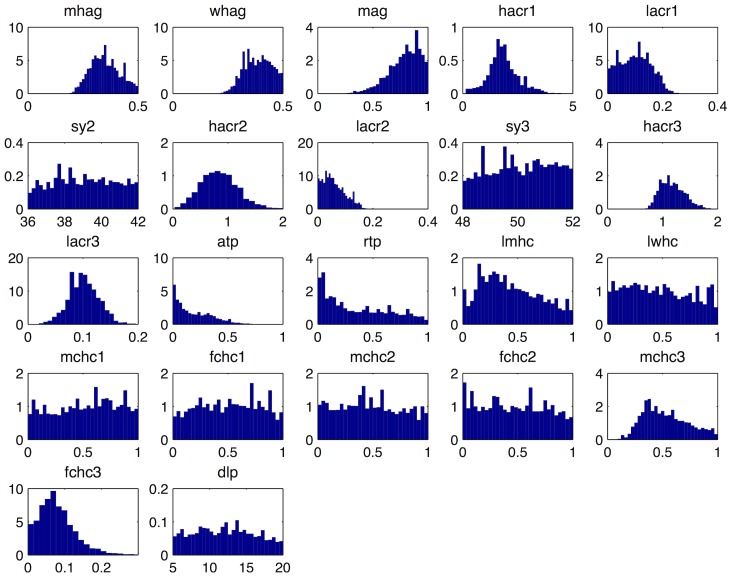
Posterior samples drawn with the importance sampling method described in section ‘Posterior Sampling’. Each panel shows the samples drawn for one of the 22 simulator inputs. Their full names and descriptions can be found in Table 1.

In this section we present the results of the method described in section ‘Posterior sampling’ for drawing approximate posterior samples from the model. The non-implausible samples at the end of wave 9 were fitted with a multivariate normal distribution. Its covariance matrix was then inflated by a factor of 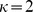 and this formed the proposal distribution 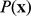. The model likelihood 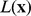 was defined as described in section ‘Posterior sampling’. Using 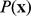, 200000 samples were proposed and their weights were calculated from the ratio 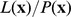. From this set of 200000 samples, 10000 samples were chosen with probability defined by their respective weights. The results are shown in Fig. 10, where the shrinkage of the input space and the particular shape the approximate posterior distribution takes for different simulator inputs is evident.

Fig. 10 also shows that the model is over-parameterised with flat posteriors over the permissible input ranges for 9 of the inputs, implying that the available empirical data were not informative for all the input parameters. Complex mechanistic simulators, such as the one studied in this paper are not designed to help us analyse a particular data set, but rather to help us understand a real world system. As such, they can include processes that we consider important for the understanding the physical system, which however, may not be identifiable from the available data. History matching helps with identifiability problems, rather than covering them up: it allows us to quantify how much information is in the data regarding the simulator's parameters and gives guidance as to what fresh data might be needed to improve our system understanding. Furthermore, it is unaffected by multiple modes or correlation ridges in the posterior, which are typical manifestations of identifiability issues and plague other calibration methods, such as those based on MCMC.

In models of the complexity and the dimensionality such as the one studied here, we would argue against reporting a single best estimate of the parameters, as there is always likely to be some uncertainty, that will be missed out by a single best estimate. Additionally, lack of identifiability does not imply that a history matched model is not useful. When using the history matched model to make a prediction, we would run it at a range of inputs over the non-implausible space, to get a range of output values. This range may still be informative (e.g. lead to the same decision). Finally, if the non-implausible set leads to an unhelpfully wide range of output values, we might then consider whether there are other calibration data available, and whether these data could be informative. The last point can be verified by looking at the related model outputs over the corresponding set of non-implausible inputs: the more the output values vary, the more informative new calibration data will be.

## Discussion

In this paper we presented a tutorial on history matching and emulation. History matching is an iterative procedure that reduces the simulator's input space by identifying areas that are unlikely to provide a good match to the empirical data. History matching relies on the efficiency of emulators, which are a Bayesian representation of the complex simulator and are parameterised iteratively during history matching.

We presented a case study where we history matched a 22 input simulator known as Mukwano, simultaneously fitting its 18 outputs. After 9 iterations of history matching, the non-implausible input space was reduced by a factor of 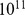. While evaluating the entire input space, the final system had a 65% probability of selecting a parameter set that fitted all 18 outputs, a percentage that could have been improved further had we continued with more iterations. This approach therefore, provides a method to generate large numbers of runs that give acceptable matches to the calibration targets, while at the same time dramatically shrinks the non-implausible input space. The Mukwano model was found to be in agreement with the observed data, various features of its structure were discussed and the region of input space corresponding to all acceptable matches was identified. A simulation study, which used a smaller version of Mukwano for validating the performance of history matching, is included in the online supplementary material.

When employing this method, Bayesian emulator construction has to be carefully implemented as otherwise the implausible space rejection rate can be small, especially if the simulator's output is not a relatively smooth function of its inputs. This condition can cause the code uncertainty (emulator's posterior variance) to be large and make it harder for the implausibility measure to reject particular input values. A more careful emulator construction, e.g. by using more detailed mean functions and more training points should increase the input space rejection rate.

History matching is thematically linked to calibration methods such as ABC or Bayesian model calibration, but has the important conceptual difference of discarding implausible areas of the input space as opposed to attempting to make probabilistic statements about the most likely input values given the empirical data. The latter methods could fail if they were applied to a large input space of a multi-input multi-output simulator. However, their application on the reduced space that is the output of history matching, could produce hybrid methods that combine the strengths of both approaches.

The method we proposed in this paper, deals with stochastic simulators assuming that the uncertainty introduced by the stochasticity of the outputs is constant at each wave with respect to the value of the inputs. This may not be the case, and knowledge of how the stochastic output variability changes with the inputs can increase the space reduction rate [39], [40]. Additionally, multi-output simulators very often exhibit correlation between their outputs, which was ignored in this work. Taking output correlation into account would improve the emulation process and subsequently the performance of history matching, but would require far more sophisticated emulators, as well as more detailed observation uncertainty and model discrepancy specifications, and hence we leave this to future work.

We conclude that history matching and emulation are useful additions to the toolbox of infectious disease modellers. Further research is required to explicitly address the stochastic nature of the simulator as well as to account for correlations between outputs.

## Supporting Information

S1 Text **Building a single output emulator.**
(PDF)

S2 Text **Simulation study.**
(PDF)

S3 Text **Terminology.**
(PDF)

## References

[1] Law A (2007) Simulation modeling and analysis. McGraw Hill.

[2] Spear RC, Hubbard A, Liang S, Seto E (2002) Disease transmission models for public health decision making: toward an approach for designing intervention strategies for Schistosomiasis Japonica. Environ Health Perspect 110: 907–915. 12204826 10.1289/ehp.02110907 PMC1240991

[3] Bauer AL, Beauchemin CAA, Perelson AS (2009) Agent-based modeling of host-pathogen systems: The successes and challenges. Information Sciences 179: 1379–1389. 20161146 10.1016/j.ins.2008.11.012 PMC2731970

[4] Grimm V, Railsback SF (2005) Individual-based Modeling and Ecology. Princeton University Press.

[5] White RG, Glynn JR, Orroth KK, Freeman E, Bakker R (2008) Male circumcision for HIV prevention in sub-saharan Africa: who, what and when? AIDS 22: 1841–1850. 18753931 10.1097/QAD.0b013e32830e0137

[6] Dewilde S, Anderson R (2004) The cost-effectiveness of screening programs using single and multiple birth cohort simulations: a comparison using a model of cervical cancer. Medical Decision Making 24: 486. 15358997 10.1177/0272989X04268953

[7] Grimm V, Berger U, Bastiansen F, Eliassen S, Ginot V (2006) A standard protocol for describing individual-based and agent-based models. Ecological Modelling 198: 115–126.

[8] May RM (2004) Uses and abuses of mathematics in biology. Science 303: 790–793. 14764866 10.1126/science.1094442

[9] Ionides EL, Bretó C, King AA (2006) Inference for nonlinear dynamical systems. Proceedings of the National Academy of Sciences USA 103: 18438–18443. 10.1073/pnas.0603181103 PMC3020138 17121996

[10] Gibson GJ, Renshaw E (1998) Estimating parameters in stochastic compartmental models using Markov chain methods. IMA Journal of Mathematics Applied in Medicine and Biology 15: 19–40.

[11] O'Neill PD, Roberts GO (1999) Bayesian inference for partially observed stochastic epidemics. Journal of the Royal Statistical Society Series A (General) 162: 121–129.

[12] Cook AR, Otten W, Marion G, Gibson GJ, Gilligan CA (2007) Estimation of multiple transmission rates for epidemics in heterogeneous populations. Proceedings of the National Academy of Sciences USA 104: 20392–20397. 10.1073/pnas.0706461104 PMC2154441 18077378

[13] Jewell CP, Kypraios T, Neal P, Roberts GO (2009) Bayesian analysis for emerging infectious diseases. Bayesian Analysis 4: 465–496.

[14] Keeling MJ, Ross JV (2008) On methods for studying stochastic disease dynamics. Journal of the Royal Society Interface 5: 171–181. 10.1098/rsif.2007.1106 PMC2705976 17638650

[15] Cauchemez S, Ferguson NM (2008) Likelihood-based estimation of continuous-time epidemic models from time-series data: Application to measles transmission in London. Journal of the Royal Society Interface 5: 885–897. 10.1098/rsif.2007.1292 PMC2607466 18174112

[16] O'Neill PD, Balding DJ, Becker NG, Eerola M, Mollison D (2000) Analyses of infectious disease data from household outbreaks by Markov chain Monte Carlo methods. Applied Statistics 49: 517–542.

[17] Cauchemez S, Valleron AJ, Boëlle PY, Flahault A, Ferguson NM (2008) Estimating the impact of school closure on influenza transmission from Sentinel data. Nature 452: 750–755. 18401408 10.1038/nature06732

[18] Toni T, Welch D, Strelkowa N, Ipsen A, Strumpf MPH (2009) Approximate Bayesian computation scheme for parameter inference and model selection in dynamical systems. Journal of the Royal Society Interface 6: 187–202. 10.1098/rsif.2008.0172 PMC2658655 19205079

[19] McKinley TJ, Cook AR, Deardon R (2009) Inference in epidemic models without likelihoods. The International Journal of Biostatistics 5..

[20] Conlan AJK, McKinley TJ, Karolemeas K, Brooks Pollock E, Goodchild AV, et al (2012) Estimating the hidden burden of bovine tuberculosis in Great Britain. PLoS Computational Biology 8: e1002730. 23093923 10.1371/journal.pcbi.1002730 PMC3475695

[21] McKinley TJ, Ross JV, Deardon R, Cook AR (2013) Simulation-based Bayesian inference for epidemic models. Computational Statistics and Data Analysis: in press.

[22] Andrieu C, Doucet A, Holenstein R (2010) Particle Markov chain Monte Carlo methods. Journal of the Royal Statistical Society, Series B (Methodological) 72: 269–342.

[23] Stout NK, Knudsen AB, Kong CY, McMahon PM, Gazelle GS (2009) Calibration methods used in cancer simulation models and suggested reporting guidelines. Pharmacoeconomics 27: 533–545. 19663525 10.2165/11314830-000000000-00000 PMC2787446

[24] Punyacharoensin N, Edmunds WJ, De Angelis D, White RG (2011) Mathematical models for the study of HIV spread and control amongst men who have sex with men. European Journal of Epidemiology 26: 695–709. 21932033 10.1007/s10654-011-9614-1

[25] Kennedy MC, O'Hagan A (2001) Bayesian calibration of computer models. Journal of the Royal Statistical Society Series B 63: 425–464.

[26] Saltelli A, Chan K, Scott EM (2000) Sensitivity analysis. Wiley Chichester.

[27] Oakley JE, O'Hagan A (2004) Probabilistic sensitivity analysis of complex models: a Bayesian approach. J R Statist Soc B 66: 751–769.

[28] Craig PS, Goldstein M, Seheult AH, Smith JA (1997) Pressure matching for hydrocarbon reservoirs: a case study in the use of Bayes linear strategies for large computer experiments. (with discussion). in Case Studies in Bayesian Statistics, eds C Gastonis, et al Springer-Verlag III: 37–93.

[29] Goldstein M, Rougier J (2009) Reified Bayesian modelling and inference for physical systems. Journal of Statistical Planning and Inference 139: 1221–1239.

[30] Goldstein M, Seheult A, Vernon I (2013) Assessing model adequacy. In: Wainwright J, Mulligan M, editors, Environmental Modelling: Finding Simplicity in Complexity, Second Edition, John Wiley & Sons, Ltd, Chichester, UK: Wiley-Blackwell.

[31] Sacks J, Welch WJ, Mitchell TJ, Wynn HP (1989) Design and analysis of computer experiments. Statistical Science 4: 409–435.

[32] Bayarri MJ, Berger JO, Paulo R, Sacks J, Cafeo JA, et al (2007) A framework for the validation of computer models. Technometrics 49: 138–154.

[33] Henderson DA, Boys RJ, Krishnan KJ, Lawless C, et al (2009) Bayesian emulation and calibration of a stochastic computer model of mitochondrial DNA deletions in substantia nigra neurons. Journal of the American Statistical Association 104: 76–87.

[34] Higdon D, Gattiker J, Williams B, Rightley M (2008) Computer model calibration using high-dimensional output. Journal of the American Statistical Association 103: 570–583.

[35] Vernon IR, Goldstein M, Bower RG (2010) Galaxy formation: a Bayesian uncertainty analysis. Bayesian Analysis 5: 619–670.

[36] Bower RG, Vernon I, Goldstein M, Benson AJ, Lacey CG, et al (2010) The parameter space of galaxy formation. MonAst 407: 2017–2045.

[37] Vernon I, Goldstein M, Bower RG (2014) Galaxy formation: Bayesian history matching for the observable universe. Statistical Science (to appear).

[38] Cumming JA, Goldstein M (2009) Bayes linear uncertainty analysis for oil reservoirs based on multiscale computer experiments. In: O'Hagan A, West M, editors, Handbook of Bayesian Analysis, Oxford, UK: Oxford University Press.

[39] Vernon IR, Goldstein M (2010) A Bayes linear approach to systems biology. Technical report, MUCM Technical Report.

[40] Vernon I, Goldstein M (2014) Bayes linear emulation and history matching of stochastic systems biology models, in preparation.

[41] Williamson D, Goldstein M, Allison L, Blaker A, Challenor P, et al (2013) History matching for exploring and reducing climate model parameter space using observations and a large perturbed physics ensemble. Climate Dynamics 41: 1703–1729.

[42] Brynjarsdottir J, O'Hagan A (2010) Learning about physical parameters: The importance of model discrepancy. Technical report, http://www.tonyogahan.co.uk/academic/pub.html.

[43] Goldstein M, Rougier JC (2006) Bayes linear calibrated prediction for complex systems. JASA 101: 1132–1143.

[44] McKay MD, Beckman RJ, Conover WJ (1979) A comparison of three methods for selecting values of input variables in the analysis of output from a computer code. Technometrics 21: 239–245.

[45] Loeppky JL, Sacks J, Welch WJ (2009) Choosing the sample size of a computer experiment: a practical guide. Technometrics 51: 366–376.

[46] Williamson D, Vernon IR (2013) Efficient uniform designs for multi-wave computer experiments.

[47] Vernon I, Goldstein M (2009) Bayes linear analysis of imprecision in computer models, with application to understanding galaxy formation. In: Augustin T, Coolen FPA, editors, ISIPTA'09: Proceedings of the Sixth International Symposium on Imprecise Probability: Theories and Applications. Durham, UK: SIPTA, pp.441–450.

[48] Rasmussen CE, Williams CKI (2006) Gaussian Processes for Machine Learning. The MIT press.

[49] Andrianakis I, Challenor P (2012) The effect of the nugget on Gaussian process emulators of computer models. Computational Statistics & Data Analysis 56: 4215–4228.

[50] Bastos LS, O'Hagan A (2009) Diagnostics for Gaussian process emulators. Technometrics 51: 425–438.

[51] Conti S, O'Hagan A (2010) Bayesian emulation of complex multi-output and dynamic computer models. Journal of Statistical Planning and Inference 140: 640–651.

[52] Rougier J (2008) Efficient emulators for multivariate deterministic functions. Journal of Computational and Graphical Statistics 17: 827–843.

[53] Fricker TE, Oakley JE, Urban NM (2013) Multivariate Gaussian process emulators with nonseparable covariance structures. Technometrics 55: 47–56.

[54] Strong M, Oakley JE, Chilcott J (2012) Managing structural uncertainty in health economic decision models: a discrepancy approach. Journal of the Royal Statistical Society, Series C 61: 25–45.

[55] Pukelsheim F (1994) The three sigma rule. The American Statistician 48: 88–91.

[56] McCreesh N, O'Brien K, Nsubuga RN, Shafer LA, Bakker R, et al (2012) Exploring the potential impact of a reduction in partnership concurrency on HIV incidence in rural Uganda: a modeling study. Sexually Transmitted diseases 39: 407–413. 22592824 10.1097/OLQ.0b013e318254c84a

[57] Mulder DW, Nunn AJ, Kamali A, Nakiyingi J, Wagner HU, et al (1994) Two-year HIV-1-associated mortality in a Ugandan rural population. Lancet 343: 1021–1023. 7909054 10.1016/s0140-6736(94)90133-3

[58] Mulder DW, Nunn AJ, Kamali A, Wagner HU, Kengeya-Kayondo JF (1994) HIV-1 incidence and HIV-1-associated mortality in a rural Ugandan population cohort. AIDS 8: 87–92. 8011241 10.1097/00002030-199401000-00013

[59] Seeley J, Wagner U, Mulemwa J, Kengeya-Kayondo J, Mulder D (1991) The development of a community-based HIV/AIDS counselling service in a rural area in Uganda. AIDS Care 3: 207–217. 1878404 10.1080/09540129108253064

[60] Santner TJ, Williams BJ, Notz WI (2003) The Design and Analysis of Computer Experiments. New York: SV.

