# Supplementary material for: Bayesian History Matching of Complex Infectious Disease Models Using Emulation: A Tutorial and a Case Study on HIV in Uganda
Source: PLoS Comput Biol. 2015 Jan 8;11(1):e1003968. doi: 10.1371/journal.pcbi.1003968 (PMC4288726; doi:10.1371/journal.pcbi.1003968)
Supplement: S1 Text — Building a single output emulator. (PDF) [file pcbi.1003968.s001.pdf]

Appendix S1: Building a single output emulator.

This document describes the procedure for building an emulator. We start with the simpler case of a zero mean-unit variance emulator and we proceed with the more general case that we follow for building the emulators used in the main manuscript.

A zero-mean, unit-variance emulator

According to their definition [1] a Gaussian process is a collection of random variables, any finite number of which have a joint Gaussian distribution.

Suppose that $u(\mathbf{x})$ is a zero mean and unit variance Gaussian process, we can write this as

$$u(\mathbf{x}) \sim \mathcal{GP}(0, c(\mathbf{x}, \mathbf{x}')). \tag{1}$$

The $c(\mathbf{x}, \mathbf{x}')$ term is known as the covariance function and plays a fundamental role in Gaussian processes as it determines the association between $u(\mathbf{x})$ and $u(\mathbf{x})'$ based on the distance between $\mathbf{x}$ and $\mathbf{x}'$. If we denote this distance as W, the covariance function can take a multitude of forms, some of the most common are shown in table 1. The covariance functions cannot take an arbitrary form, as they have to satisfy the constraint of positive definiteness (see chapter 4 of [1]). Suppose that we have two p-dimensional points $\mathbf{x} = [x_1, x_2, \ldots, x_p]$ and $\mathbf{x}' = [x'_1, x'_2, \ldots, x'_p]$. Their weighted distance is defined as

Correlation function	Formula
Gaussian	$c(\mathbf{x}, \mathbf{x}') = \exp\left(-W^2\right)$
α-exponential ($\alpha < 2$)	$c(\mathbf{x}, \mathbf{x}') = \exp\left(-W^\alpha\right)$
Matérn 3/2,	$c(\mathbf{x}, \mathbf{x}') = \left(1 + \sqrt{3}W\right) \exp\left(-\sqrt{3}W\right)$

Table 1. Some common correlation functions $c(\mathbf{x}, \mathbf{x}')$, with W denoting a weighted distance between $\mathbf{x}$ and $\mathbf{x}'$.

$$W = \left[\sum_{i=1}^{p} \left(\frac{x_i - x'_i}{\delta_i}\right)^2\right]^{1/2} \tag{2}$$

The parameters $\delta = [\delta_1, \delta_2, \ldots, \delta_p]$ are known as *correlation lengths* and weigh the distance between $\mathbf{x}$ and $\mathbf{x}'$. A large δ_i implies that $u(\mathbf{x})$ and $u(\mathbf{x}')$ will be strongly correlated along the ith dimension of $\mathbf{x}$ and vice versa.

Suppose now that we observe the Gaussian process at n discrete points $D = \{\mathbf{x}_i : i = [1, \ldots, n]\}$ and we get the vector of observations $u(D) = [u(\mathbf{x}_1), u(\mathbf{x}_2), \ldots, u(\mathbf{x}_n)]$. D and $u(D)$ here represent the emulator's training points. According to the definition of a Gaussian process, the observations $u(D)$ will follow a joint Gaussian distribution,

$$p(u(D)|\delta) = \frac{|\tilde{A}|^{-1/2}}{(2\pi)^{n/2}} \exp\left[-\frac{1}{2} u(D)^{\mathrm{T}} \tilde{A}^{-1} u(D)\right], \tag{3}$$

where $\tilde{A}$ is the covariance matrix of the design points $u(D)$, which is defined as

$$\tilde{A} = \begin{pmatrix} 1 & c(\mathbf{x}_1, \mathbf{x}_2) & \dots & c(\mathbf{x}_1, \mathbf{x}_n) \\ c(\mathbf{x}_2, \mathbf{x}_1) & 1 & \dots & c(\mathbf{x}_2, \mathbf{x}_n) \\ \vdots & \vdots & \ddots & \vdots \\ c(\mathbf{x}_n, \mathbf{x}_1) & \dots & \dots & 1 \end{pmatrix}.$$

The distribution of $u(D)$ is conditioned on the covariance function hyperparameters δ. One way of training the emulator could be to search for a value of δ that maximises equation 3, i.e.

$$\hat{\delta} = \arg\max_{\delta} \left[p(u(D)|\delta)\right]$$

A more involved approach, would be to define a prior distribution for δ and draw samples from the posterior distribution $p(\delta|u(D))$. For sake of simplicity, we assume here the first approach.

We are now interested in estimating the value of the Gaussian process at an unknown point $\mathbf{x}$, conditional on the observed data $u(D)$ and $\hat{\delta}$. This distribution is

$$p(u(\mathbf{x})|u(D), \hat{\delta}) = \mathcal{N}(\mathrm{E}^*[u(\mathbf{x})], \mathrm{Var}^*[u(\mathbf{x})]) \tag{4}$$

where

$$\begin{aligned} \mathrm{E}^*[u(\mathbf{x})] &= c(\mathbf{x})^{\mathrm{T}} \tilde{A}^{-1} u(D) \\ \mathrm{Var}^*[u(\mathbf{x})] &= c(\mathbf{x}, \mathbf{x}') - c(\mathbf{x})^{\mathrm{T}} \tilde{A}^{-1} c(\mathbf{x}') \end{aligned}$$

with the vector $c(\mathbf{x})$ defined as $c(\mathbf{x}) = [c(\mathbf{x}, \mathbf{x}_1), c(\mathbf{x}, \mathbf{x}_2), \cdots, c(\mathbf{x}, \mathbf{x}_n)]^{\mathrm{T}}$. The above distribution is the posterior distribution of a zero mean - unit variance emulator.

The general case

We will now present the equations of the more general non-zero mean emulator with arbitrary variance. The model assumed is

$$g(\mathbf{x}) \sim \mathcal{GP}(h^{\mathrm{T}}(\mathbf{x})\beta, \sigma^2 c(\mathbf{x}, \mathbf{x}')). \tag{5}$$

which is the same as equation 1, apart from the mean term $h^{\mathrm{T}}(\mathbf{x})\beta$ and σ^2 that controls the scaling (variance) of the process. Assuming again a set of n observations $g(D)$, the likelihood of this model is

$$\begin{aligned} p(g(D)|\beta, \sigma^2, \theta_c) &= \mathcal{N}(H\beta, \sigma^2 A) \\ &= \frac{|A|^{-1/2}}{(2\pi\sigma^2)^{n/2}} \exp\left[-\frac{1}{2\sigma^2}(g(D) - H\beta)^{\mathrm{T}} A^{-1} (g(D) - H\beta)\right] \end{aligned} \tag{6}$$

where

$$H = [h(\mathbf{x}_1), h(\mathbf{x}_2), \cdots, h(\mathbf{x}_n)]^{\mathrm{T}}$$

The matrix A is defined as $A = \tilde{A} + \nu\mathcal{I}$, where $\mathcal{I}$ a diagonal matrix and ν is a parameter, commonly known as the nugget [2]. The role of the nugget is to account for the existence of noise in the model (i.e. if the observations $g(D)$ were noisy measurements of the process, rather than the process itself), while it can also safeguard against numerical instabilities that can occur in the calculations involved in training and fitting the emulator. The parameters δ and ν are jointly referred to as $\theta_c = [\delta, \nu]$.

If we assume a non informative prior for σ^2 and β $p(\sigma^2, \beta) \propto \sigma^{-2}$ [3], these two parameters can be marginalised in the Bayesian sense, yielding

$$p(g(D)|\theta_c) \propto \frac{|A|^{-1/2}|H^{\mathrm{T}}A^{-1}H|^{-1/2}}{(\hat{\sigma}^2)^{(n-q)/2}} \tag{7}$$

where $\hat{\sigma}^2 = g(D)^{\mathrm{T}}[A^{-1} - A^{-1}H(H^{\mathrm{T}}A^{-1}H)^{-1}H^{\mathrm{T}}A^{-1}]g(D)$. This was the expression we optimised w.r.t. θ_c when we trained the emulators in the manuscript.

In the same fashion, one can obtain the posterior distribution for the emulator $p(g(\mathbf{x})|g(D), \hat{\theta}_c)$, which will be a multivariate t-distribution, with $n - q$ degrees of freedom and mean

$$\mathrm{E}^*[g(\mathbf{x})] = h^{\mathrm{T}}(\mathbf{x})\hat{\beta} - c^{\mathrm{T}}(\mathbf{x})A^{-1}(g(D) - H\hat{\beta}) \tag{8}$$

where $\hat{\beta} = (H^{\mathrm{T}}A^{-1}H)^{-1}H^{\mathrm{T}}A^{-1}g(D)$ and variance

$$\mathrm{Var}^*[g(\mathbf{x})] = \frac{\hat{\sigma}^2}{n-q-2}c_1(\mathbf{x}, \mathbf{x}'), \tag{9}$$

with

$$\begin{aligned} c_1(x, x') &= c(x, x) - c^{\mathrm{T}}(x)A^{-1}c(x) \\ &+ (h^{\mathrm{T}}(x) - c^{\mathrm{T}}(x)A^{-1}H)(H^{\mathrm{T}}A^{-1}H)^{-1}(h^{\mathrm{T}}(x) - c^{\mathrm{T}}(x)A^{-1}H)^{\mathrm{T}}. \end{aligned}$$

If the degrees of freedom $(n - q)$ of the t-distribution are sufficiently large (e.g. $n - q > 20$) we can consider $p(g(\mathbf{x})|g(D), \hat{\theta}_c)$ as being a multivariate normal distribution with the same mean and variance as above.

References

1. Rasmussen CE, Williams CKI (2006) Gaussian Processes for Machine Learning. The MIT press.

2. Andrianakis I, Challenor P (2012) The effect of the nugget on Gaussian process emulators of computer models. Computational Statistics & Data Analysis 56: 4215-4228.

3. Kennedy MC, O'Hagan A (2001) Bayesian calibration of computer models. Journal of the Royal Statistical Society Series B 63: 425-464.
