# Supplementary material for: Bayesian History Matching of Complex Infectious Disease Models Using Emulation: A Tutorial and a Case Study on HIV in Uganda
Source: PLoS Comput Biol. 2015 Jan 8;11(1):e1003968. doi: 10.1371/journal.pcbi.1003968 (PMC4288726; doi:10.1371/journal.pcbi.1003968)
Supplement: S2 Text — Simulation study. (PDF) [file pcbi.1003968.s002.pdf]

Appendix S2: Simulation study.

1 Introduction

In this section we present a simulation study using a smaller version of the Mukwano simulator, varying 3 of its 22 inputs, keeping the rest fixed, and matching 2 of its 18 outputs. We generate synthetic data using a known input configuration $\mathbf{x}_0$ and determine the simulator's input space that leads to an acceptable match between the output and the synthetic calibration data. Table 1 shows the variable inputs, their ranges, and the values we use to create the simulated data denoted by $\mathbf{x}_0$. Table 2 shows the outputs we match, and the mean and variance of the model output obtained from $K = 100$ runs using the input $\mathbf{x}_0$. The emulators we use here are identical to those used in the case study. We also assume zero model discrepancy i.e. $V_\delta = 0$.

We consider two different noise scenarios: in the first we use $\hat{g}(\mathbf{x}_0)$ as the calibration target and $\hat{s}(\mathbf{x}_0)$ as the uncertainty (variance) around it. We then look for all inputs $\mathbf{x} \in \mathcal{X}_1$ such that *any individual* output $f(\mathbf{x})$ (i.e. not just the mean $g(\mathbf{x})$) could be a match to the calibration data. This is equivalent to the approach we took in the case study, where we linked the physical process y to individual model runs $f(\mathbf{x})$ and not to their ensemble averages. In the second scenario we match simulator output means: that is, we consider again $\hat{g}(\mathbf{x}_0)$ as the calibration data, but this time with a variance of $\hat{s}(\mathbf{x}_0)/K$. We then look for inputs $\mathbf{x} \in \mathcal{X}_2$ such that the simulator's mean output $\hat{g}(\mathbf{x})$ matches $\hat{g}(\mathbf{x}_0)$ within the tolerance implied by the variance $\hat{s}(\mathbf{x}_0)/K$. The second noise scenario imposes far stronger restrictions on the simulator's input space and we expect its non-implausible volume $\mathcal{X}_2$ to be smaller and actually a subset of $\mathcal{X}_1$.

2 Noise scenario 1

The link we consider between the calibration data $z \equiv \hat{g}(\mathbf{x}_0)$ and the mean simulator's output $g(\mathbf{x}^*)$ at the best input $\mathbf{x}^*$ is

$$z = g(\mathbf{x}^*) + \phi + \epsilon$$

where $\mathrm{Var}[\phi] \equiv V_o = \hat{s}(\mathbf{x}_0)$ and $\mathrm{Var}[\epsilon] \equiv V_s$ set, as in the case study, equal to the 90th percentile of the sample variances $\hat{s}(\mathbf{x}_i)$ (see section 2.5). In each wave, the simulator is run at 70 different input values,

60 of which are used for training and 10 for validation. Additionally, at each wave, any simulator runs from previous waves that were non-implausible are also included in the training data. The number of runs per input point (K) are kept fixed to $K = 100$.

After 4 waves, the non-implausible space was reduced to 9% of the original. The emulator was evaluated at a further 70 input configurations drawn at random from the wave 4 non-implausible space, and 90% of these runs provided a good match to the calibration data. Figure 1 shows the outputs from the runs that were calculated as non-implausible across the 4 + 1 waves, using the criterion of section 3.5 in the main text. The red triangle is $\hat{g}(\mathbf{x}_0)$ and the shaded patch represents 3 standard deviations calculated using the variance $\hat{s}(\mathbf{x})$. The green dots show the simulator run $f(\mathbf{x})$ for each non-implausible $\mathbf{x}$ that was closest to the calibration data $\hat{g}(\mathbf{x}_0)$.

Figure 2 shows the distribution of the non-implausible space at wave 4 in the form of 2 dimensional minimum implausibility and optical depth projections. This figure also shows the distribution of the non-implausible simulator runs, mentioned above, as black dots. The input calibration point $\mathbf{x}_0$ is shown as a triangle. The figure shows that all the simulator runs that matched the calibration data fall within the calculated non-implausible space. Additionally, the model is non-identifiable for this set of inputs and outputs and this noise levels. History matching however, identifies all parts of the input space that can lead to a match, and is not affected by how complicated this space may be.

3 Noise scenario 2

In this section we match the mean simulator output by dividing the uncertainties V_s and V_o by K = 100. Everything else remains the same as in noise scenario 1. After 12 waves the non-implausible space is 0.7% of the original and 70 further simulator evaluations at points drawn from the non-implausible space of wave 12 matched the calibration data at a rate of 91%.

Figure 3 shows the outputs from all the non-implausible runs plotted against the calibration data $\hat{g}(\mathbf{x}_0)$ and the 3 standard deviation error bars implied by the variance $\hat{s}(\mathbf{x})/K$. The green dots now show the mean simulator outputs $\hat{g}(\mathbf{x})$ for all the non-implausible simulator runs generated under this noise scenario.

Figure 4 shows the non-implausible space at wave 12 using minimum implausibility and optical depth plots. It also shows the calibration target $\mathbf{x}_0$ and the location of all the non-implausible runs, shown in

figure 3, as black dots. It is clear the simulator can match the calibration data, even with the narrow uncertainty intervals we used in this case, for a multitude of input configurations, which are not necessarily close to the input $\mathbf{x}_0$ we used to generate the calibration data.

4 Discussion

The simulation study showed that the simulator was capable of matching the calibration data with several input configurations that were not necessarily close to the calibration inputs $\mathbf{x}_0$, implying that the simulator was non-identifiable. This posed no problem to the history matching methodology, which identified a non-implausible space that resulted in 'good' simulation runs 90% of the time, after 4 and 12 waves for the 2 noise scenarios. The input space could be restricted further by adding more outputs that are informative about the 3 inputs we studied.

Number	Input description	Abbr.	Min.	Max.	$\mathbf{x}_0$
1	Proportion of men in the high sexual activity group	*mhag*	0.01	0.5	0.35
2	High activity contact rate (risk behaviour 1) [partners/yr]	*hacr1*	0	10	2.07
3	Low activity contact rate (risk behaviour 1) [partners/yr]	*lacr1*	0	0.4	0.18

Table 1. Simulator inputs used in the simulation study, initial ranges, and values used for generating the synthetic calibration data.

Number	Output description	Abbr.	$\hat{g}(\mathbf{x}_0)$	$\hat{s}(\mathbf{x}_0)$
1	HIV prevalence in 1992 (male)	$p92m$	0.11	$5.7 \cdot 10^{-4}$
2	HIV prevalence in 1992 (female)	$p92f$	0.13	$7.9 \cdot 10^{-4}$

Table 2. Simulator outputs used in the simulation study, showing also the mean ($\hat{g}(\mathbf{x}_0)$) and variance ($\hat{s}(\mathbf{x}_0)$) of the synthetic calibration data.

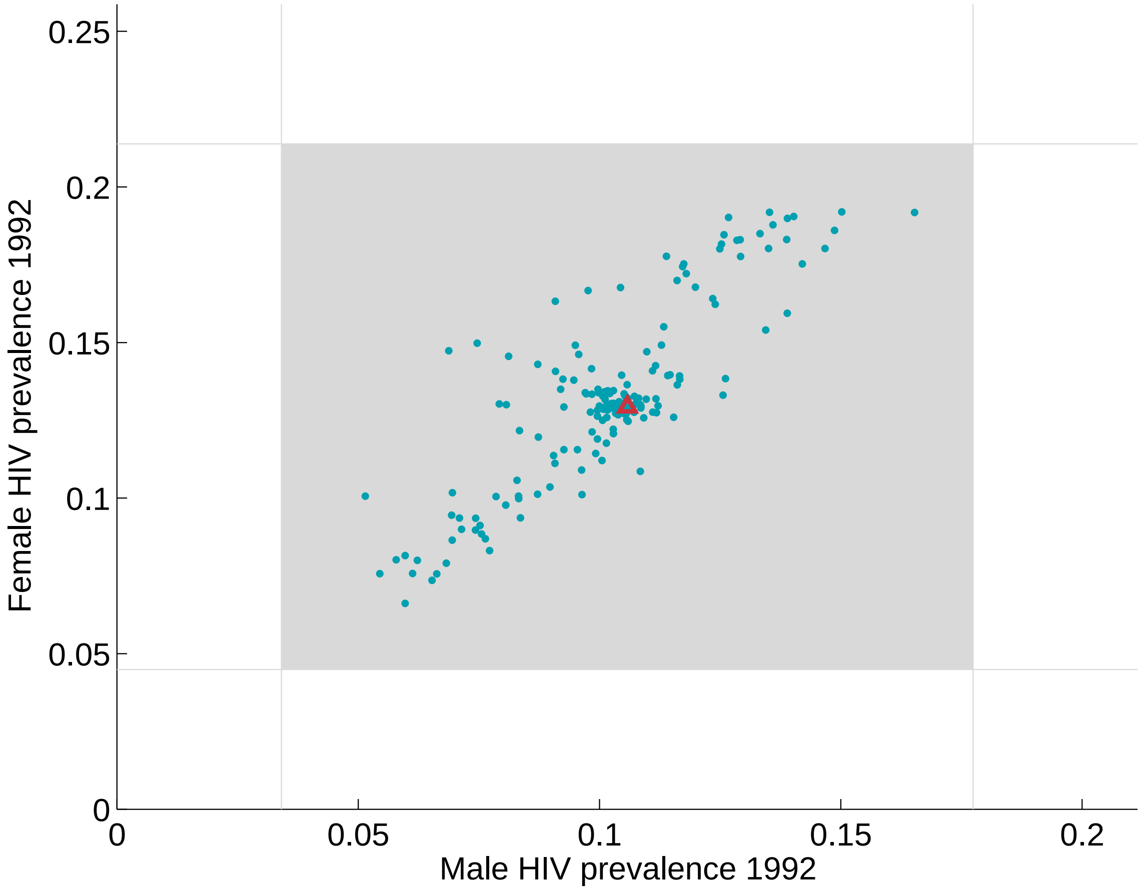

Figure 1. Output of the non-implausible simulator runs. The triangle is the calibration target and the shaded area represents 3 standard deviations. The green dots are the individual simulator runs that best matched the calibration data, from all the simulator runs that were non-implausible.

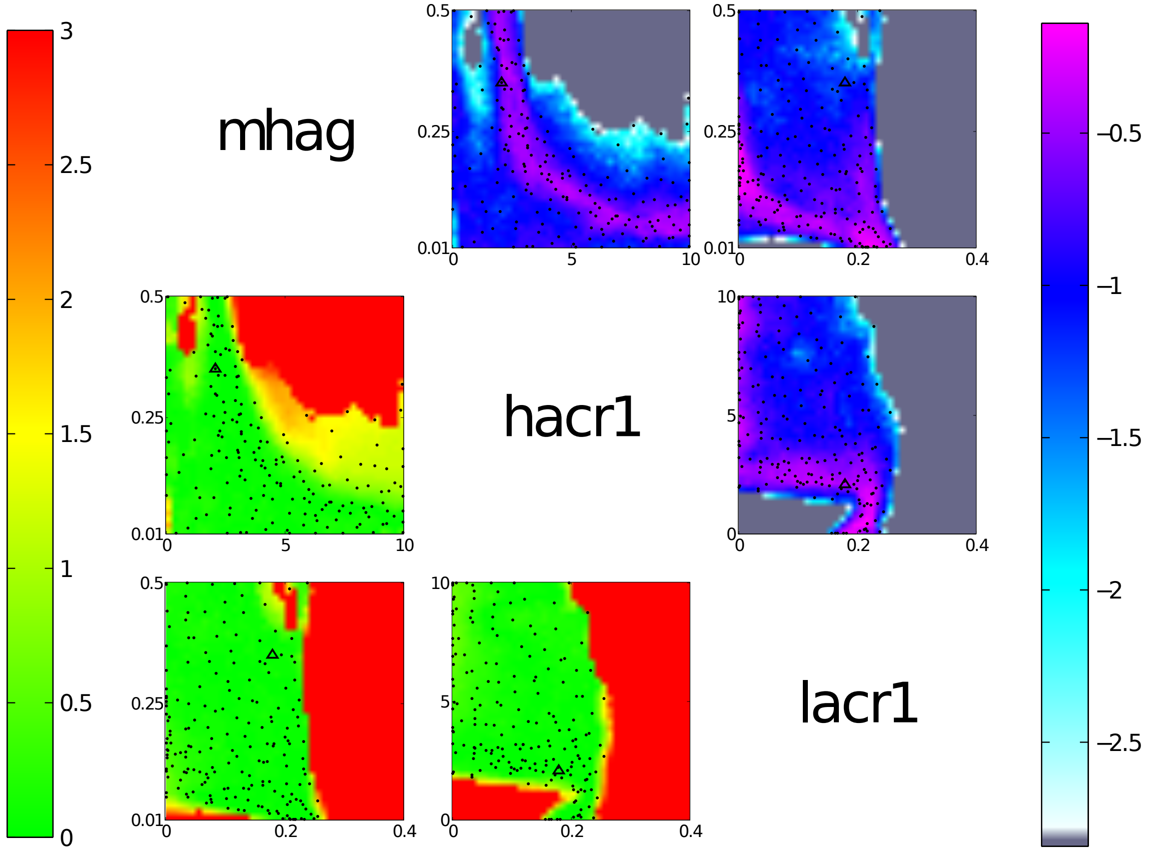

Figure 2. Minimum implausibility (lower triangle) and optical depth plots (upper triangle). The black dots show the position of the non-implausible simulator runs and the triangle shows the input $\mathbf{x}_0$ used for generating the calibration data.

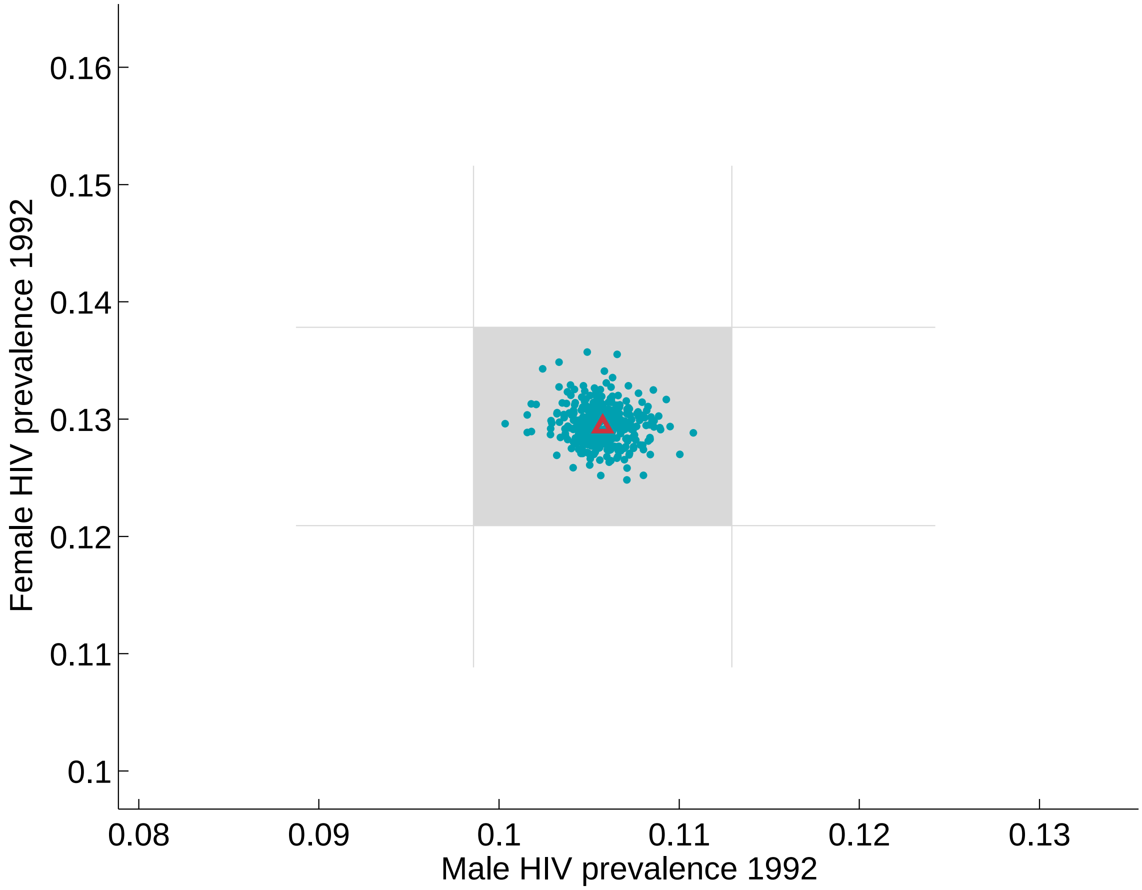

Figure 3. Output of the non-implausible simulator runs (noise scenario 2). The triangle is the calibration target and the shaded area represents 3 standard deviations. The green dots are the mean simulator outputs from all the simulator runs that were non-implausible.

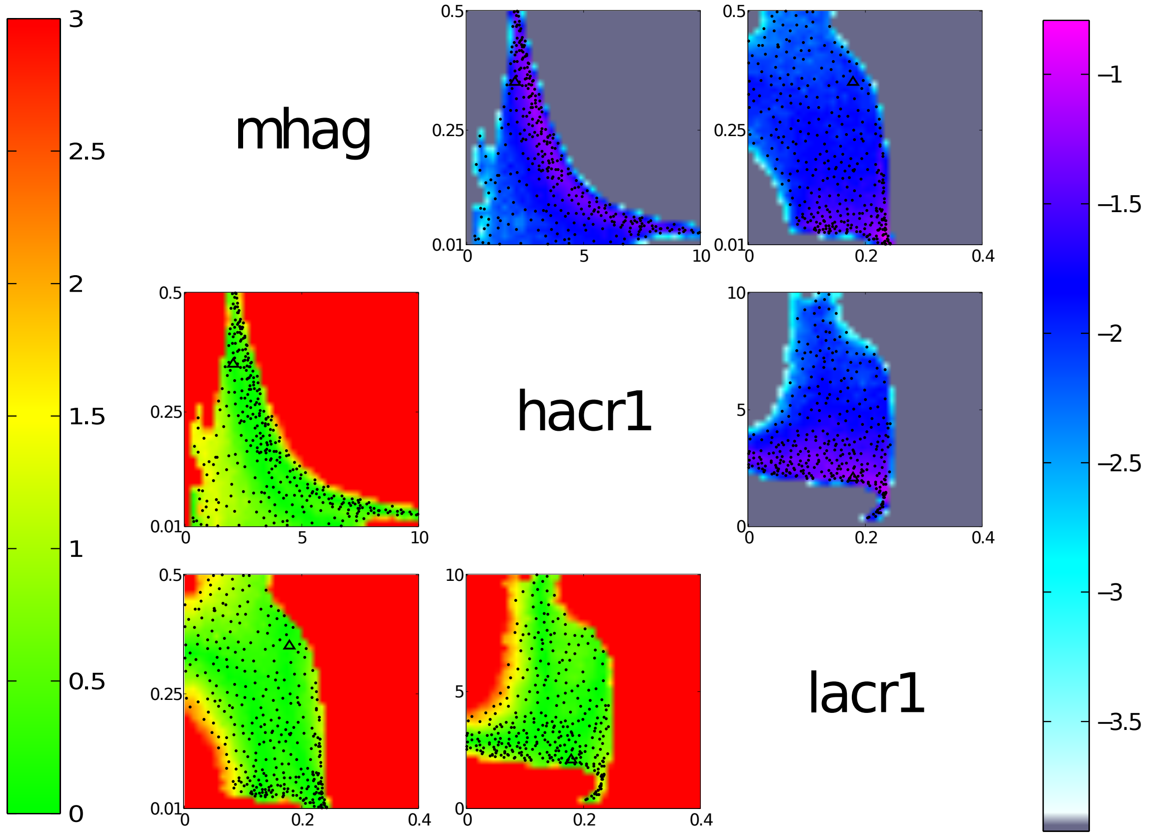

Figure 4. Minimum implausibility (lower triangle) and optical depth plots (upper triangle) (noise scenario 2). The black dots show the position of the non-implausible simulator runs and the triangle shows the input $\mathbf{x}_0$ used for generating the calibration data.
