# Supplementary material for: Bayesian History Matching of Complex Infectious Disease Models Using Emulation: A Tutorial and a Case Study on HIV in Uganda
Source: PLoS Comput Biol. 2015 Jan 8;11(1):e1003968. doi: 10.1371/journal.pcbi.1003968 (PMC4288726; doi:10.1371/journal.pcbi.1003968)
Supplement: S3 Text — Terminology. (PDF) [file pcbi.1003968.s003.pdf]

Terminology

Simulator The computer model.

Stochastic simulator A simulator whose output(s), for a particular input value, are draws from an (unknown) distribution.

Emulator A statistical representation of the simulator, which is usually fast to evaluate.

Implausibility A measure of whether a simulator input is likely to result in output(s) that will match the target data, which when large suggests that the input will not match the data.

Calibration The search for sets of simulator input values that result in outputs that fit the target data, sometimes also taken to mean the inference about the true value of the model parameters.

History matching Iterative method that identifies and removes parts of the input space unlikely to result in a match between the simulator output and the target data.

Observation uncertainty Uncertainty introduced due to inaccuracies in measurements made of the physical process.

Code uncertainty Uncertainty arising from the inability to run the simulator using every input configuration, due to limited computational resources.

Model discrepancy Discrepancy between the simulator output evaluated at appropriate choices of input, and the physical process.

Ensemble variability Output variability due to the simulator being stochastic.
